# Size-Selective FET Sensors Based on Semiconducting Single-Walled Carbon Nanotubes and Metal–Organic Frameworks

**DOI:** 10.1021/acsami.6c06870

**Published:** 2026-05-18

**Authors:** Zidao Zeng, Samia Afrin, Gefan He, Haitao Liu, Nathaniel L. Rosi, Alexander Star

**Affiliations:** † Department of Chemistry, 6614 University of Pittsburgh, Pittsburgh, Pennsylvania 15260, United States; ‡ Department of Chemical & Petroleum Engineering, University of Pittsburgh, Pittsburgh, Pennsylvania 15260, United States; § Department of Bioengineering, University of Pittsburgh, Pittsburgh, Pennsylvania 15260, United States; ∥ Clinical and Translational Science Institute, University of Pittsburgh, Pittsburgh, Pennsylvania 15260, United States

**Keywords:** MOF thin-film, PVDF thin-film, capacitance-modulation FETs, opioid sensing, dopamine sensing

## Abstract

This study presents a universal method for fabricating size-selective, liquid-gated field-effect transistor (FET) sensors using semiconducting single-walled carbon nanotubes (scSWCNTs) combined with metal–organic frameworks (MOFs) and poly­(vinylidene fluoride) (PVDF) polymer barriers. By employing a layer-by-layer architecture, scSWCNTs were coated with a single layer of MOF crystals followed by a PVDF thin film, which restricts the access to scSWCNTs to only through MOF pores. This design enables a gate capacitance modulation mechanism, where only analyte molecules small enough to enter the MOF pores can modulate the scSWCNT conductance. Four MOFs, namely UiO-66, UiO-67, ZIF-8, and MIL-96, were fabricated into the scSWCNT/MOF/PVDF FET sensors and tested for their sensing capabilities toward norfentanyl (NF) and dopamine (DA) in 0.1 M KCl solution. UiO-67 devices showed positive responses to NF due to favorable pore size matching, while devices fabricated with other MOFs exhibited negative responses due to pore size exclusion. Tests with DA also confirmed the size-selective sensing abilities of the sensors. Long-term stability tests revealed that weak interactions between MOFs and PVDF limit the sensor durability in aqueous solution. Despite this limitation, the proposed approach shows considerable potential for constructing diverse size-selective sensors that enhance specificity and selectivity in scSWCNT/MOF/polymer sensing platforms.

## Graphical abstract

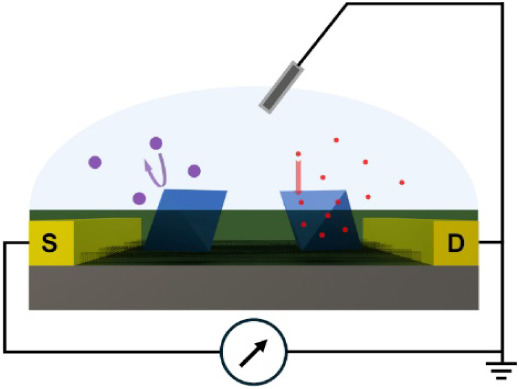

## 1 Introduction

Metal–organic frameworks (MOFs) are a class of crystalline porous materials constructed from metal nodes and multidentate organic linkers. Their exceptionally high surface area, tunable pore sizes, and chemically functionalizable internal surfaces make them ideal candidates for molecular recognition and chemical sensing.^1^ Through engineering of pore size and ligand functionality, MOFs can be tailored to facilitate specific host–guest interactions, which are essential for selective analyte detection.^2^ Consequently, MOFs have been widely incorporated into gravimetric (e.g., quartz crystal microbalance) and photoluminescent sensing platforms, where analyte adsorption induces measurable mass or optical changes.^3–5^

Among various sensing methodologies, electrochemical and electronic sensors, including chemiresistors and field-effect transistors (FETs), are gaining popularity due to their high sensitivity, compact form factors, and ease of operation.^6^ These attributes make electronic and electrochemical sensors ideal candidates for point-of-care and out-of-lab sensor applications.^7^ However, the application of MOFs in these platforms is less common due to their intrinsically low electrical conductivity. In typical MOFs, metal centers are usually isolated by insulating organic ligands, resulting in the absence of free charge carriers and efficient charge transport pathways.^8^ To enable electrochemical and electronic applications, conventional MOFs are commonly deposited as thin films onto the electrical transducers that correspond to specific sensing modalities, for instance, glassy carbon electrodes (GCEs) for conventional electrochemical sensors or semiconducting metal oxide thin films for chemiresistive sensors. These integration strategies inevitably inherit the limitations of their underlying detection methodologies. For example, metal-oxide-based sensors typically require elevated operating temperatures,^9–11^ whereas most MOF-based electrochemical sensors are limited to detecting redox-active analytes.^12–15^ Recent research has explored alternative approaches, including the incorporation of π-conjugated ligands and/or redox-active metal nodes, to impart electrical conductivity to MOFs themselves.^16,17^ However, such approaches often require specific ligand–metal combinations, constraining the broader structural and chemical tunability that makes MOFs attractive as sensor materials.

With advances in sensor research, MOFs have been increasingly utilized as selective layers to enhance the selectivity and sensitivity of chemiresistive sensors. Jo et al. demonstrated that spin-coating a thin mixed-matrix membrane (MMM), comprising ZIF-7 and polyether block amide, onto TiO_2_ thin-film sensors significantly enhanced their selectivity toward formaldehyde over ethanol.^18^ Park et al. developed dual-MOF-layered chemiresistive sensors composed of a conductive MOF (cMOF) sensing layer and an additional functional MOF overlayer. By depositing this additional MOF thin film onto the cMOF via solution shearing, the selectivity or sensitivity of the cMOF sensor could be improved based on the intrinsic properties of the chosen overlayer MOF.^19^ Beyond chemiresistive sensing applications, MOFs have also been integrated into FET sensors. Kumar et al. reported a selective ethanol sensor by *in situ* growth of a surface-mounted MOF (SURMOF) on graphene-based FETs (GFETs).^20^ Keum et al. introduced a size-selective silicon FET biosensor capable of discriminating physiological small molecules by modifying the extended gate with a thin layer of a 2D cMOF.^21^ This modification was achieved through a layer-by-layer MOF assembly facilitated by a self-assembled monolayer. Current integration methods typically require MOFs to be fabricated into thin films through complex or labor-intensive methods, adding complexity to the device fabrication process and limiting the broader application of diverse MOFs.

A less explored strategy to utilize MOFs as sensing material is combining MOFs with single-walled carbon nanotubes (SWCNTs). SWCNTs stand out as excellent electrical transducers for chemical sensing due to their excellent long-range electrical conductivity and single-layer atomic structure, which makes them highly sensitive to local chemical potential variations.^22^ Semiconducting SWCNTs (scSWCNTs), in particular, are favorable for sensing applications, as the absence of a density of states near the Fermi level enhances their sensitivity to charge perturbations.^23^ Previous studies have demonstrated that sensors fabricated from scSWCNTs exhibit superior sensitivity compared to those using mixtures of scSWCNTs and metallic SWCNTs (m-SWCNTs).^24–28^ SWCNTs have diameter- and chirality-dependent electronic properties, and as-synthesized SWCNT samples inherently contain a mixture of approximately two-thirds scSWCNTs and one-third m-SWCNTs due to the lack of control in producing SWCNTs with well-defined structures.^29^ Recent advances in carbon nanotube postsynthesis sorting techniques now provide commercially available scSWCNTs with purities exceeding 99.9%.^30,31^

Despite the conceptual promise of SWCNT–MOF hybrids, physically mixing SWCNTs with MOFs fails to synergize the selective adsorption in MOFs with the electronic transduction capabilities of SWCNTs. Our previous work has shown that true hybridization into composite material is essential.^32–34^ MOFs can be heterogeneously nucleated and grown directly on the SWCNT surface, either through coordination with carboxylic defects on nanotubes or via π–π stacking interactions between aromatic MOF ligands and graphitic SWCNT sidewalls.^32,33^ The intrinsic porous structure of MOFs provides unique local environments around the nanotubes, enabling SWCNTs to leverage MOF’s inherent size-selectivity and molecular affinity. For example, our group has successfully demonstrated size-based discrimination of chemically similar carbohydrates using Cu_2_(HHTP)_2_/SWCNT composites,^35^ and size-based detection of norfentanyl using UiO-67/SWCNT composites.^36^ Furthermore, by integrating the hydrogen-adsorbing MOF, HKUST-1, with palladium nanoparticle-functionalized scSWCNTs, we achieved a detection limit of 70 ppb for hydrogen gas.^37^

However, fabricating conductive SWCNT/MOF composites for chemical sensing remains technically challenging. Achieving heterogeneous MOF growth on SWCNT surfaces requires meticulous optimization of synthesis conditions. Moreover, the increased ionic strength from metal ions in MOF precursor solutions hinders effective SWCNT dispersion, complicating composite synthesis. Additionally, the significant dimensional mismatch between SWCNTs (typically around 1 nm in diameter and micrometers in length) and MOF crystals (typically hundreds of nanometers to several micrometers in diameter) complicates the formation of conductive networks, especially when nanotube surfaces are mostly embedded within MOF crystals. Furthermore, commercially available scSWCNTs are often wrapped in π-conjugated chirality-selective polymers,^30^ which limit their dispersibility in solvents commonly used for MOF assembly, thus restricting composite synthesis to mixed nanotube types with reduced sensing performance.

To overcome these limitations, we developed a universal method for constructing size-selective, liquid-gated FET sensors using MOFs and commercially available scSWCNTs without the need to form a composite. Instead, we employed a modular layer-by-layer device architecture: an scSWCNT network is initially deposited onto prefabricated interdigitated electrodes (IDEs) to form the conductive transducer layer. A monolayer of presynthesized MOF crystals is then deposited as a size-selective layer. Lastly, a thin film of poly­(vinylidene fluoride) (PVDF) is spin-coated onto the device, embedding the MOF nanocrystals and effectively blocking direct access to the scSWCNT surface. In this configuration, only analyte molecules small enough to diffuse into the MOF pores can alter the local chemical environment, which, in turn, modulates the conductance of the underlying scSWCNT network, enabling size-selective sensing.

In this work, four MOFs, namely UiO-66, UiO-67, ZIF-8, and MIL-96, were successfully fabricated into scSWCNT/MOF/PVDF FET sensors and evaluated for their sensing performance toward norfentanyl (NF) and dopamine (DA). These selected MOFs feature distinct metal centers, metal–ligand chemistries, and pore sizes. The chemical diversity among these MOFs was leveraged to demonstrate the versatility and general applicability of the reported sensor fabrication method. NF is a primary metabolite of fentanyl, an acute synthetic opioid.^38^ Due to the ongoing opioid epidemic crisis in the United States, NF is an important biomarker for monitoring fentanyl exposure, particularly because it is present in higher concentrations in urine and offers a prolonged detection window.^39,40^ Dopamine, on the other hand, is a neurotransmitter and a clinically significant biomarker associated with various neurological disorders such as schizophrenia, Alzheimer’s disease, and Parkinson’s disease.^41–43^ In this study, NF and DA served as representative molecules of different sizes to assess and probe the sensing capabilities of the sensors fabricated with the selected MOFs.

## 2 Results and Discussion

### 2.1 Sensor Device Fabrication

The sensor devices were fabricated in three steps, as shown in Figure 1: (1) a random network of scSWCNTs was deposited on prefabricated gold interdigitated electrodes (IDEs) using dielectrophoresis. (2) A single layer of presynthesized MOF crystals was deposited on top of the scSWCNT as the size-selective layer. (3) A hydrophobic PVDF film was spin-coated to block nonselective pathways for analyte molecules. The PVDF polymer was used as the blocking layer due to its documented compatibility with various MOFs.^44,45^ Previous reports showed that PVDF can form mixed matrix membranes (MMMs) with different MOFs, with less infiltration into MOF pores compared to alternatives like polyethylene glycol (PEO).^46^

**Figure 1:**
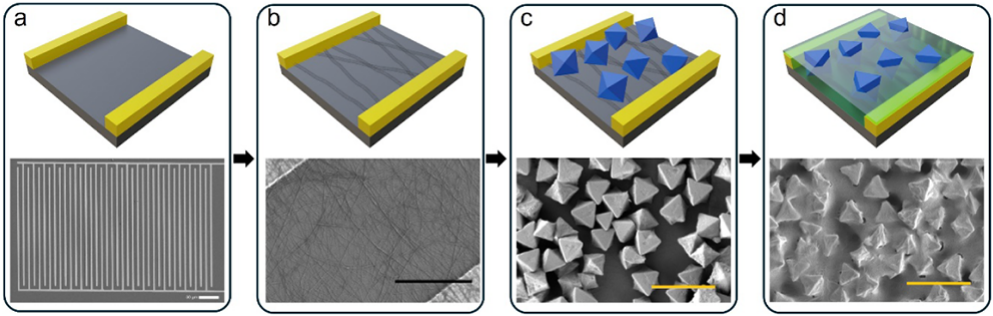
Schematic illustration of the sensor fabrication process and corresponding SEM images of the device after each step. (a) Interdigitated electrode (IDE) device. (b) scSWCNT deposition. (c) Single-layer UiO-67 nanocrystal deposition. (d) PVDF polymer spin-coating. The scale bar in a is 30 μm. Scale bars in b, c, and d are 2 μm.

The fabrication process was initially investigated with UiO-67. To achieve a single-layer MOF deposition, UiO-67 was suspended in 1-butanol via bath sonication, and the resulting suspension was drop-cast onto the water surface of a Petri dish. The surface tension at the 1-butanol/water interface facilitated the formation of a uniform, single-layered film of UiO-67 nanocrystals (Figure S1).^47^ After 1-butanol evaporation, silicon dies predeposited with scSWCNT were lifted through the floating MOF film to achieve transfer. Cross-sectional SEM images were taken to confirm the successful transfer of the single-layer MOF (Figure 2a). After activating the transferred UiO-67 in a vacuum oven, a layer of PVDF (5 wt % in DMF) was spin-coated on the wafer inside a glovebox (H_2_O < 0.1 ppm). The PVDF films were subsequently air-dried overnight in the glovebox, followed by baking in an oven to remove residual DMF. The scSWCNT/MOF/PVDF sensors were last activated in a vacuum oven. The 5 wt % PVDF solution in DMF was optimized based on reported recipes for PVDF/MOF mixed-matrix membranes.^44,45^ The PVDF concentration was reduced from 7.5 wt % to obtain a thin coating layer that partially embedded the MOF crystals (Figure S2). When a 7.5 wt % PVDF solution was used, the MOF crystals were fully encapsulated within the PVDF layer. As a result, ion transport through the MOF pores was obstructed, leading to a diminished gating effect in the scSWCNT device compared to devices prepared with 5 and 2.5 wt % PVDF solutions (Figure S3). Before testing with an analyte, the sensor devices were incubated in 0.1 M KCl for at least 24 h to wet the pores in the MOFs. SEM images were taken at each step to verify the successful MOF deposition and proper PVDF coating (Figure 1b–d). A cross-sectional SEM image confirmed the successful embedment of UiO-67 nanocrystals in the PVDF matrix (Figure S4). Low-magnification SEM images corresponding to Figure 1C and 1D were taken to show the coating uniformity over a larger area (Figure S5). It was necessary to spin-coat the PVDF film under a humidity-controlled environment. When the PVDF thin film was coated under ambient conditions, major macrovoids on the PVDF film were observed because of phase separation caused by water absorbed from the air (Figure S6).^48^ It is noteworthy that there were still some minor macrovoids between UiO-67 and PVDF when spin-coating was performed under dry conditions because of the lack of covalent interactions between these two components.

**Figure 2:**
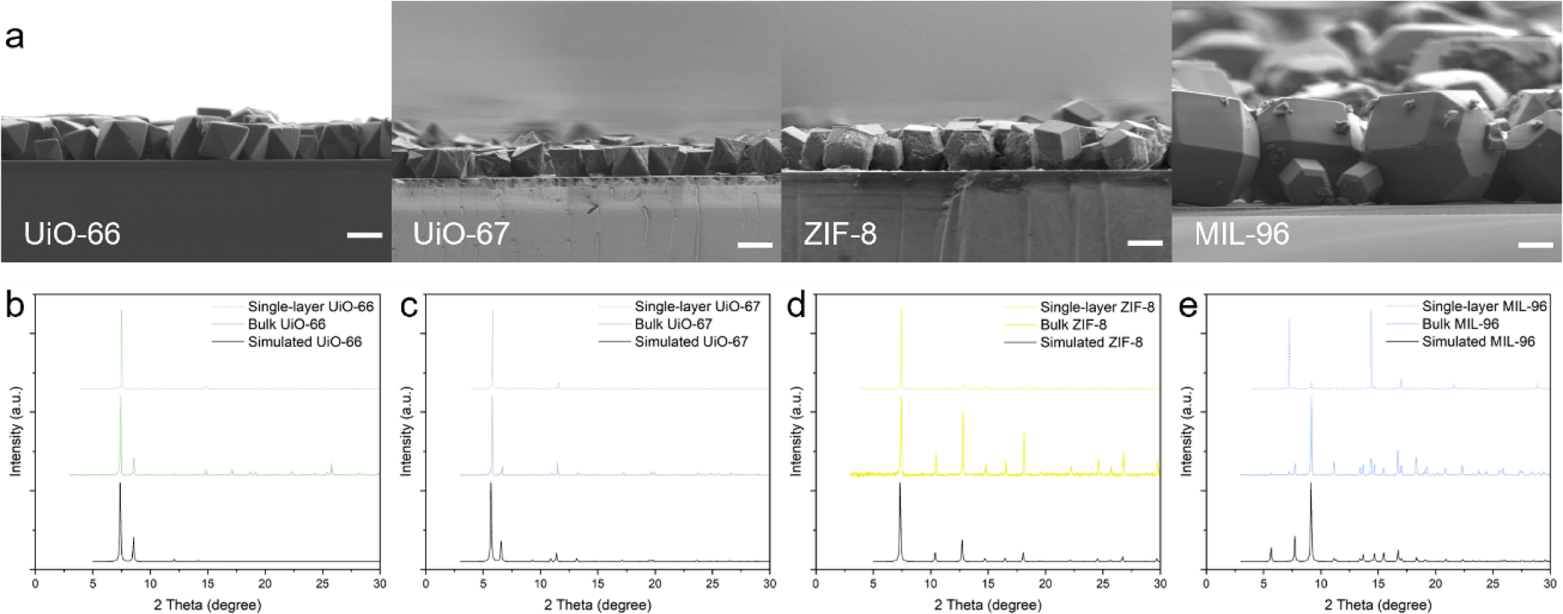
(a) Cross-section SEM images of transferred single-layer MOF on a silicon wafer. Scale bars are 1 μm. XRD patterns of bulk and single-layer MOFs for (b) UiO-66, (c) UiO-67, (d) ZIF-8, and (e) MIL-96.

### 2.2 Norfentanyl Sensing

Four types of MOFs, namely UiO-66 (pore diameter 8.6 Å),^36^ UiO-67 (pore diameter 13.0 Å),^36^ ZIF-8 (pore diameter 11.6 Å),^49^ and MIL-96 (pore diameter 8.8 Å),^50^ were employed in this study. They were synthesized according to established protocols. Characterization of these MOFs by transmission electron microscopy (TEM) and X-ray diffraction (XRD) confirmed their morphology and crystallinity (Figure S7 and Figure 2b–e). The XRD peak ratio of the single-layer MOF differed from that of the bulk powder and simulated pattern due to the preferred orientation alignment of the MOF crystals at the water interface.^47^ Nitrogen adsorption isotherms were measured to assess their porosity (Figure S8). UiO-66, UiO-67, and ZIF-8 exhibited typical type-I adsorption/desorption curves, with Brunauer–Emmett–Teller (BET) surface areas calculated to be 1396 m^2^ g^–1^ for UiO-66, 2647 m^2^ g^–1^ for UiO-67, and 1648 m^2^ g^–1^ for ZIF-8. MIL-96 showed minimal nitrogen uptake at 77 K, consistent with the previous literature result.^50^

The fabricated devices were tested as liquid-gated FETs in a 0.1 M KCl solution. An Ag/AgCl reference electrode was placed in contact with the gating liquid to serve as the gate electrode (Figure 3a). UiO-67 was first evaluated as the selective layer for norfentanyl (NF) detection as a proof of concept. Previous studies from our group have demonstrated good size-matching between UiO-67 pores and NF molecular dimensions when investigating MOF-nanotube composites.^36^ NF solutions were prepared in 0.1 M KCl at concentrations ranging from 1 ppb to 100 ppm and were used as the gating liquid for testing. Four different sensor architectures were tested: (1) scSWCNT, (2) scSWCNT/UiO-67 (single-layer UiO-67 on scSWCNTs), (3) scSWCNT/PVDF, and (4) scSWCNT/UiO-67/PVDF. At ambient conditions, scSWCNTs behave as p-type semiconductors because of the doping effect of adsorbed oxygen.^51^ When exposed to NF, the bare scSWCNT devices exhibited a shift to negative voltages in the FET transfer characteristics, i.e., drain-source current vs gate voltage characteristic (*I*_ds_–*V*_g_), indicative of NF-induced n-doping (Figure 3b). The observed n-doping is consistent with the charge transfer from the electron-donating piperidine rings of the adsorbed NF molecules. A decrease in *I*_ds_ can be observed with increasing NF concentration, as electron transfer reduces the main carrier (hole) density in the p-type scSWCNTs. In contrast, the scSWCNT/UiO-67/PVDF devices displayed increased *I*_sd_ with increasing NF concentration (Figure 3c), featuring a symmetrical bending on both the p- and n-branches, indicating NF-induced gate capacitance modulation rather than doping (Figure 3d).

**Figure 3:**
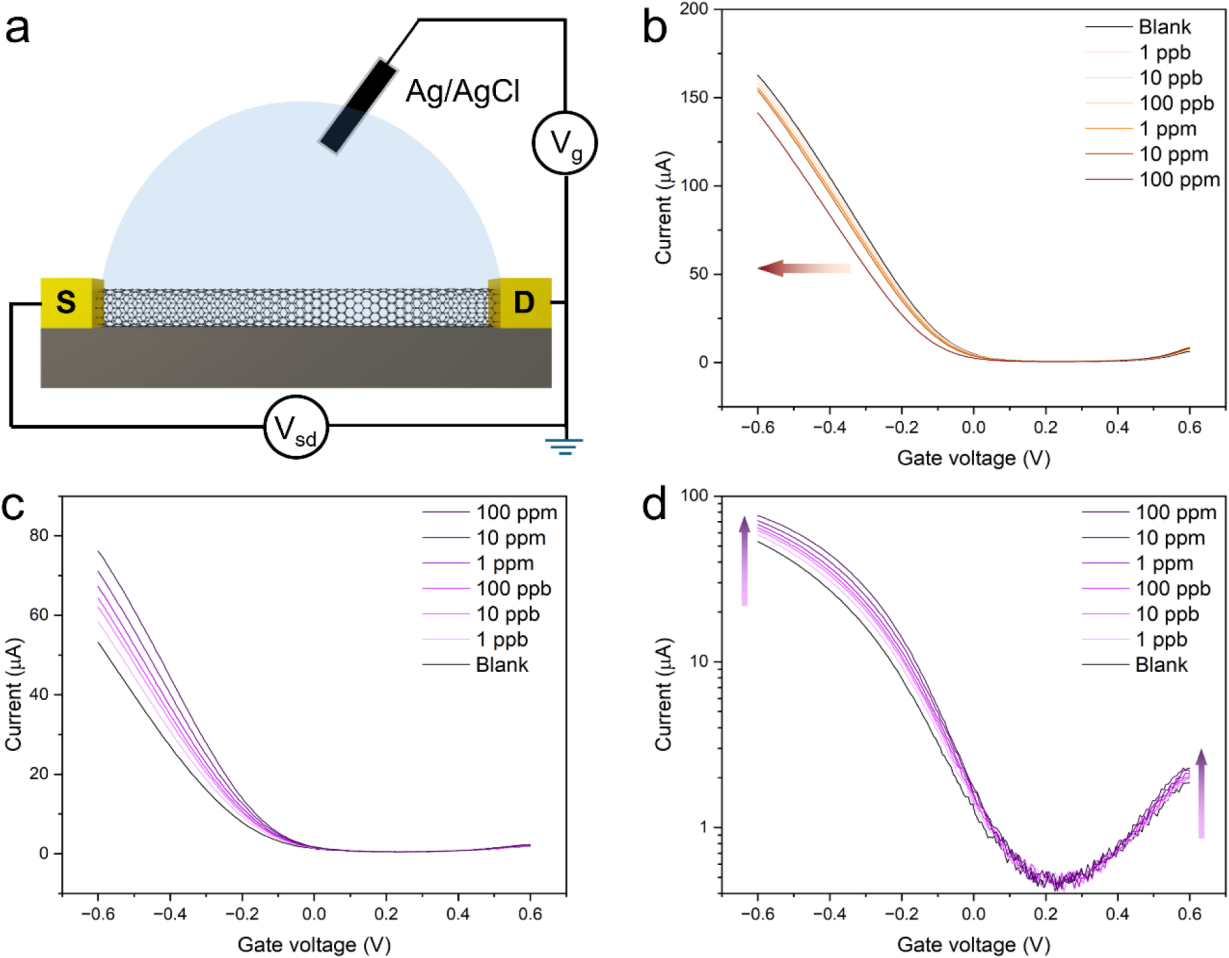
(a) Schematic illustration of the liquid-gated FET testing setup. (b) FET transfer characteristics, i.e., source–drain current versus gate voltage (*I*_ds_–*V*_g_) curves, of a typical scSWCNT device exposed to norfentanyl at different concentrations in 0.1 M KCl. (c, d) FET transfer characteristics of the scSWCNT/UiO-67/PVDF device when exposed to norfentanyl at different concentrations in 0.1 M KCl, plotted on a linear (c) and a logarithmic (d) scale.

The sensor responses were quantified using the equation: $R=\frac{\Delta I}{I_{0}}$ at −0.2 *V*_g_, where *I*_0_ is the source–drain current measured in the blank sample (0.1 M KCl), and Δ*I* is the difference between the current measured in the test sample and that in the blank. The gate voltage of −0.2 V was selected for calculating the sensor response, as a comparison with responses derived at alternative gate voltages (Figure S9) showed better response magnitude and linearity. A calibration plot was constructed by plotting the relative response against norfentanyl concentration on a logarithmic scale. As shown in Figure 4a, bare scSWCNT devices displayed a concentration-dependent decrease in signal, whereas scSWCNT/UiO-67/PVDF devices exhibited a concentration-dependent increase. The scSWCNT/PVDF devices demonstrated negligible sensitivity to NF, confirming that PVDF alone did not contribute to the sensing signal and served as an effective barrier, which prevented NF molecule adsorption on scSWCNT surfaces. scSWCNT/UiO-67 devices exhibited a slight increase in current at lower NF concentrations (1 ppb–10 ppb), followed by a gradual decrease in current with increasing NF concentration. This pattern was attributed to the adsorption of NF molecules on scSWCNT surfaces. The doping effect from NF resulted in a continuous negative shift of the *I*_sd_–*V*_g_ curve (Figure S10). scSWCNT/UiO-67/PVDF devices fabricated using PVDF solutions with different weight percentages were evaluated. Devices prepared with 5 wt % PVDF exhibited the highest sensing response toward norfentanyl (Figure S11).

**Figure 4:**
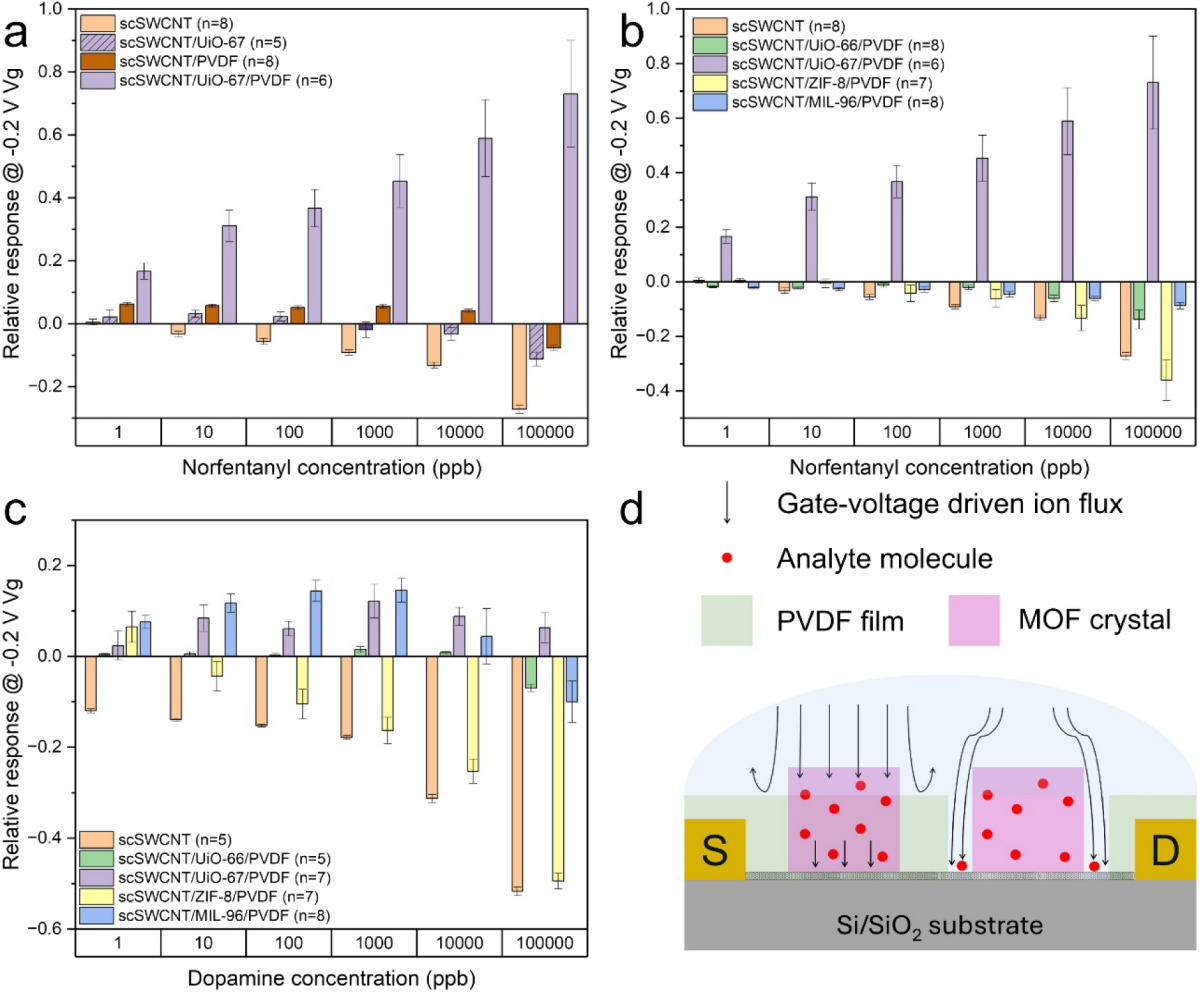
Calibration plot of different scSWCNT/MOF/PVDF devices. (a) Calibration plot of different device architectures fabricated with UiO-67 and their responses toward norfentanyl in 0.1 M KCl solution. (b) Calibration plot of scSWCNT/MOF/PVDF devices toward norfentanyl in 0.1 M KCl solution. (c) Calibration plot of scSWCNT/MOF/PVDF devices toward dopamine in 0.1 M KCl solution. (d) Schematic illustration of size-selective sensing achieved through the gate capacitance modulation mechanism.

The opposing responses observed between scSWCNT/UiO-67/PVDF devices and bare scSWCNT devices indicated that these two architectures operate via different sensing mechanisms. The scSWCNT/UiO-67/PVDF devices functioned through gate capacitance modulation, which occurred only when access to the scSWCNTs was restricted to the UiO-67 pores. In this scenario, NF molecules were adsorbed and trapped within the MOF pores, altering the local gate capacitance experienced by scSWCNTs. In liquid-gated FETs, the device operation relies on gate voltage-driven ion flux to form an electrical double layer that gates the transducer layer. When ions travel through the pores of UiO-67, the presence of trapped NF molecules hinders the ion flux, resulting in a change in gate capacitance. Interestingly, the experimentally observed symmetrical bending of both p- and n-branches of the *I*–*V*_g_ curves appeared inconsistent with theoretical predictions. Typically, one would anticipate a decrease in device conductance due to less effective gating from the ion-blocking effect, as observed in our previous studies on SWCNT/MOF composites.^35,36^ However, as neither lateral shifting nor asymmetric tilting was evident and instead symmetrical bending was observed, we attributed the conductance change to modulation of local capacitance. In contrast, without the PVDF barrier, the gate-voltage-driven ion flux bypasses the MOF channels and directly gates the underlying CNTs, thereby nullifying the gate capacitance change within the MOF pores, as depicted in Figure 4d.

To further support the proposed capacitance modulation mechanism, cyclic voltammetry (CV) experiments were performed on three device architectures: scSWCNT, scSWCNT/UiO-67, and scSWCNT/UiO-67/PVDF. The source and drain electrodes were shorted to use the deposited scSWCNT network as the working electrode, while a platinum wire and an Ag/AgCl electrode served as the counter and reference electrodes, respectively. CV measurements were conducted from +0.6 V to −0.6 V (vs Ag/AgCl) at a scan rate of 50 mV s^–1^. A 0.1 M KCl solution, with and without norfentanyl (1–100 ppb), was used as the electrolyte (Figure S12). In the absence of redox-active species, the measured current is dominated by capacitive charging. Capacitance was evaluated at −0.2 V to align with the voltage used for the FET response. The capacitance at −0.2 V was calculated by dividing the average current at this potential by the scan rate. To account for device-to-device variation arising from scSWCNT deposition, the capacitance values were normalized to those measured in blank 0.1 M KCl. With increasing norfentanyl concentration, the capacitance increased in the scSWCNT/UiO-67/PVDF device, whereas it decreased in the scSWCNT and scSWCNT/UiO-67 devices (Figure S13). These trends are consistent with the conductance changes observed in the transfer characteristics, supporting a sensing mechanism governed by capacitance modulation in the scSWCNT/UiO-67/PVDF device.

The limit of detection (LOD) of the sensor devices was calculated using the equation $\mathrm{LOD}=\frac{3S_{y}}{S}$ , where *S*_*y*_ is the standard deviation of residuals (i.e., standard error of regression) and *S* is the slope of the calibration curve (Figure S15a). Based on this method, the LOD of the scSWCNT/UiO-67/PVDF devices for norfentanyl was determined to be 12 ppb.

To assess the general applicability of the sensor fabrication approach, additional MOFs (UiO-66, ZIF-8, and MIL-96) were similarly fabricated and tested. SEM imaging confirmed successful single-layer MOF deposition and satisfactory PVDF integration (Figure 2a and Figure S16). However, when exposed to NF, devices based on these MOFs behaved similarly to those of bare scSWCNT devices due to their smaller pore sizes, preventing NF adsorption (Figure 4b). No gate capacitance modulation was observed among these devices. Instead, NF molecules traveled through the PVDF film via the macrovoids between MOFs and PVDF, doping the underlying scSWCNTs, which causes the *I*_sd_–*V*_g_ characteristic to shift to negative gate voltage (Figure S17). It is noteworthy that without the PVDF film, scSWCNT/MOF devices responded toward NF in a similar way as bare scSWCNT devices, except in the case of UiO-67 (Figure S18). This difference can be attributed to the fact that NF can be adsorbed in UiO-67 from the gating liquid, which reduces the concentration of NF that the scSWCNTs are exposed to. To further validate this hypothesis, five more layers of UiO-67 MOF were deposited onto the scSWCNT/UiO-67/PVDF devices. The resulting responses from these devices were lower than those of regular scSWCNT/UiO-67/PVDF devices (Figure S19).

### 2.3 Dopamine Sensing

To demonstrate the efficacy of sensor devices incorporating smaller-pore MOFs, dopamine (DA) was used as a small analyte. According to the structure and the length of the C–C bond, the size of a dopamine molecule was estimated to be 10 Å × 6 Å, which is smaller than the pore diameter of UiO-66, UiO-67, ZIF-8, and MIL-96. As shown in Figure 4c, bare scSWCNT devices exhibited pronounced DA-induced doping, likely due to the strong adsorption of DA on scSWCNTs. In contrast to NF testing, devices with UiO-66, UiO-67, and MIL-96 displayed gate capacitance modulation at low DA concentrations (1 ppb–1 ppm), resulting in increased current. Similar symmetrical bending of p- and n-branches can be seen in the typical *I*_sd_–*V*_g_ curves for these three devices (Figure S20). However, at higher DA concentrations (≥10 ppm), a reversal of the responses was observed. This overturn of the sensing signal is attributed to DA molecules passing the PVDF film through the macrovoids, n-doping the scSWCNTs. The doping effect overwhelmed the effect of gating capacitance modulation. A shift to negative gate voltage in the *I*_sd_–*V*_g_ curve can be observed at 10 and 100 ppm for these three devices. scSWCNT/ZIF-8/PVDF presented unique behavior, initially showing gate capacitance modulation at 1 ppb DA concentration but reverting to doping-like behavior at higher concentrations. Post-test SEM analyses revealed that ZIF-8 degraded upon DA exposure, creating MOF-sized defects in the PVDF film (Figure S21c). In contrast, UiO-66, UiO-67, and MIL-96 retained their structural integrity (Figure S21). Post-test XRD further confirmed that the crystalline structure of UiO-66, UiO-67, and MIL-96 remained intact (Figure S22). The dissolution of ZIF-8 caused the gate capacitance modulation to fail. Without the porous MOF structure to trap DA molecules, DA attached to the scSWCNTs, causing a decrease in current through n-doping. A continuing negative shift can be observed from the typical *I*_sd_–*V*_g_ curves of scSWCNT/ZIF-8/PVDF devices (Figure S20c).

Due to the reversal of the sensing signal at higher dopamine concentrations, the LOD values for scSWCNT/MOF/PVDF devices were determined from the calibration plots in the 1 ppb–1 ppm concentration range for UiO-66, UiO-67, and MIL-96 (Figure S15b–d). The best gate voltages for these systems were selected based on the optimal dopamine sensing responses identified from measurements at alternating gate voltages (Figure S14). The resulting LODs were 158 ppb for scSWCNT/UiO-66/PVDF, 10 ppb for scSWCNT/UiO-67/PVDF, and 64 ppb for scSWCNT/MIL-96/PVDF.

### 2.4 Long-Term Sensor Stability

Long-term stability was assessed by storing devices in a 0.1 M KCl solution for two months prior to DA testing. As shown in Figure S23, UiO-66- and UiO-67-based devices showed doping-like responses, attributed to PVDF detachment from the UiO MOFs, causing major macrovoids in the film, as evidenced by SEM images (Figure S24a–b). MIL-96 devices retained their original response characteristics during this storage period. Interestingly, the initially unstable ZIF-8 devices showed improved stability during testing, displaying gate capacitance modulation. SEM imaging showed that after storage in the KCl solution, there was a layer of flake structure deposited on the surface of ZIF-8 and MIL-96 devices (Figure S24c–d). We speculated that this layer of structure protected ZIF-8 from unzipping when exposed to DA, making it possible for ZIF-8 devices to retain their porous structure and function through the gate capacitance modulation mechanism. Overall, the prolonged stability of the sensor devices was not ideal in aqueous solution, largely due to the weak interaction between MOF crystals and the PVDF film and the hydrophobic nature of PVDF. PVDF was attached to the MOF crystal through noncovalent interactions, which cannot withstand the long-term exposure to an aqueous solution. The detachment led to the formation of macrovoids between UiO MOFs and the PVDF film, disabling the size selectivity provided by MOF pores. Future improvements in scSWCNT/MOF/polymer sensor device stability could be pursued through covalent bonding to enhance polymer–MOF interactions.

Interference studies were also conducted by evaluating the sensor response toward norfentanyl in synthetic urine (SU) and bovine serum albumin (BSA) solutions (Figure S25). The device response toward norfentanyl was significantly diminished in SU, likely because of small molecular species in SU occupying the MOF pores and hindering analyte access. In contrast, a comparable response toward low concentrations of norfentanyl was retained in BSA-containing media (1 wt % in 0.1 M KCl), consistent with limited pore accessibility for larger biomolecules, supporting the proposed size-selective mechanism.

## 3 Conclusion

This study demonstrated a universal method for constructing size-selective liquid-gated FET sensors by using scSWCNT transducers integrated with single-layer MOFs and PVDF polymer barriers. These sensors operate through a gate capacitance modulation mechanism, wherein the PVDF layer effectively restricts direct access to the scSWCNTs, forcing gate voltage-driven ion flux to travel through the MOF pores. The intrinsic porosity of the MOFs acts as a sieving layer, allowing only analytes smaller than the MOF pore size to enter, subsequently altering the gate capacitance of the FET devices and modulating the scSWCNT conductance. Four different MOFs, namely UiO-66, UiO-67, ZIF-8, and MIL-96, featuring diverse metal centers, metal–ligand chemistries, and pore sizes, were successfully fabricated into size-selective liquid-gated FET sensors. The sensors were evaluated by using NF and DA solutions in 0.1 M KCl. UiO-67-based devices demonstrated positive responses toward NF due to favorable pore size matching, whereas UiO-66-, ZIF-8-, and MIL-96-based devices exhibited negative responses, as NF was too large to be adsorbed by their pores. When tested with the smaller analyte DA, devices fabricated with UiO-66, UiO-67, and MIL-96 exhibited positive responses, indicating successful gate capacitance modulation. In contrast, devices made with ZIF-8 failed to demonstrate size-selective sensing toward DA due to the degradation of the ZIF-8 framework upon exposure to DA. Long-term aqueous stability remains a challenge because of weak interactions between the MOFs and the PVDF polymer layer. Nevertheless, this fabrication approach separated the SWCNTs from the MOF synthesis process, offering a facile, modular and versatile method to incorporate MOFs into electrochemical sensors. This approach allows researchers to fully exploit advancements in MOF engineering and SWCNT alignment techniques, thereby improving the sensing performance of scSWCNT/MOF/polymer FET devices. Furthermore, this fabrication approach holds considerable promise for developing sensor arrays by employing diverse MOFs, providing a viable pathway to transform scSWCNT/MOF/polymer FET sensors into a versatile sensing platform technology.

## 4 Experimental Section

### 4.1 Chemicals and Materials

Zirconium­(IV) chloride anhydrate (Sigma-Aldrich), terephthalic acid (Sigma-Aldrich), biphenyl-4,4′-dicarboxylic acid (Sigma-Aldrich), acetic acid (Sigma-Aldrich), zinc acetate anhydrate (Sigma-Aldrich), 2-methylimidazole (Sigma-Aldrich), aluminum chloride hexahydrate (Sigma-Aldrich), 1,3,5-benzenetricarboxylic acid (Sigma-Aldrich), poly­(vinylidene fluoride) (*M*_w_ ∼ 534,000) (Sigma-Aldrich), methanol (Fisher Scientific), isopropanol (Fisher Scientific), *N*,*N*-dimethylformamide (Fisher Scientific), n-butanol (TCI Chemicals), and semiconducting-enriched SWCNT (IsoSol-S100, Raymor Industries Inc.) were purchased and used without further purification.

### 4.2 Synthesis of UiO-66 ^52^

209.7 mg of ZrCl_4_ (0.9 mmol) and 149.5 mg of terephthalic acid (0.9 mmol) were dissolved in 60 mL of DMF with bath sonication, followed by the addition of 8.24 mL of glacial acetic acid (144 mmol). The resulting solution was transferred to a 100 mL Teflon-lined autoclave and heated at 120 °C for 24 h. After cooling down to room temperature, the precipitate was collected by centrifugation and was washed three times each with DMF and MeOH. The sample then underwent a solvent exchange in MeOH at 60 °C for 3 days. The resulting white precipitate was collected by filtration, dried at 120 °C for 3 h, and stored in a desiccator.

### 4.3 Synthesis of UiO-67 ^53^

First, 38.7 mg of biphenyl-4,4′-dicarboxylic acid (BPDC) was added to 4 mL of DMF in a 4-dram vial to prepare a 0.04 M solution. The suspension was lightly sonicated and heated in an oil bath at 120 °C to facilitate dissolution, then cooled to room temperature. In an 8-dram vial, 23.3 mg of ZrCl_4_ (0.1 mmol) was dissolved in 11.19 mL of DMF and 1.31 mL of glacial acetic acid. The mixture was sonicated for 1 min and then heated in an oil bath at 120 °C for 10 min, followed by the addition of 2.5 mL of the 0.04 M BPDC solution. The resulting mixture was heated at 120 °C for 5 h. The precipitate was collected by centrifugation and washed three times each with DMF and MeOH. After undergoing a solvent exchange in MeOH for 3 days, the product was filtered, dried at 120 °C for 3 h, and stored in a desiccator.

### 4.4 Synthesis of ZIF-8 ^32^

First, 1.23 g of 2-methylimidazole (2-mIM) was dissolved in 10 mL of nanopure water to prepare a 1500 mM solution. Separately, 68.8 mg of zinc acetate was dissolved in 5 mL of nanopure water to create a 75 mM solution. In an 8-dram vial, 3 mL of water, 8 mL of the 1.5 M 2-mIM solution, and 4 mL of the 75 mM Zn­(OAc)_2_ solution were combined. The mixture was sonicated for 1 min and then left undisturbed for 3 h, resulting in a cloudy suspension. The precipitate was collected by centrifugation and washed three times, each with water and MeOH. The sample was stored in MeOH. Prior to weighing, ZIF-8 was filtered and dried at 120 °C for 3 h.

### 4.5 Synthesis of MIL-96 ^54^

In an 8-dram vial, 315 mg of AlCl_3_·6H_2_O, 315 mg of 1,3,5-benzenetricarboxylic acid (BTC), and 180 mg of NaOH were combined with 15 mL of nanopure water. The suspension was sonicated for 5 min before being transferred into a 25 mL Teflon-lined autoclave. The sealed autoclave was then heated in an oven at 150 °C for 72 h. After cooling to room temperature, the precipitate was collected by centrifugation and washed three times with DMF. The resulting white precipitate was resuspended in 10 mL of DMF and transferred to an 8-dram vial. The suspension was then heated in an oil bath with magnetic stirring at 150 °C for 24 h to remove unreacted ligands. The precipitate was collected by centrifugation and washed three times each with DMF and MeOH. After undergoing a solvent exchange in MeOH for 3 days, the final precipitate was collected by filtration, dried at 120 °C for 3 h, and stored in a desiccator.

### 4.6 Deposition of scSWCNT

Eight gold IDE devices, each measuring 300 × 200 μm with a 6 μm channel length, were fabricated on a 7 × 7 mm silicon wafer die using a conventional metal deposition and liftoff process. scSWCNTs were deposited onto these prefabricated gold IDEs through dielectrophoresis. A Keithley 3900 Arbitrary Waveform Generator provided a sine wave (10 V_pp_, 100 kHz) during deposition. 10 μL of scSWCNT suspension (0.02 mg/mL in toluene) was drop-cast onto the wafer die, and the electrical sine wave was applied across the electrodes for 2 min. The wafer was subsequently rinsed with isopropanol and dried using nitrogen gas. This deposition–wash–dry sequence was repeated two to three times to ensure sufficient CNT deposition. Finally, the devices were annealed at 200 °C for 1 h.

### 4.7 Single-Layer MOF Deposition

Specified amounts of MOF powder (4 mg for UiO-66, 2 mg for UiO-67, 4 mg for ZIF-8, and 10 mg for MIL-96) were individually dispersed in 100 μL of n-butanol inside 1.5 mL sample vials by bath sonication. 40 μL of the MOF suspension was then carefully drop-cast onto the water surface contained within a 60 mm diameter Petri dish. Upon evaporation of n-butanol, a uniform floating MOF film formed on the water surface. This floating film was subsequently transferred onto silicon dies that were predeposited with scSWCNTs, by gently lifting the dies through the MOF film. After transfer, the MOF-coated dies were allowed to air-dry and were then baked at 120 °C for 1 h.

### 4.8 PVDF Thin-Film Spin-Coating

Silicon wafer dies predeposited with scSWCNTs and single-layered MOFs were activated in a vacuum oven at 150 °C for 3 h to remove adsorbed moisture. Following activation, the wafer dies were promptly transferred into a glovebox (H_2_O < 0.1 ppm). Inside the glovebox, 20 μL of PVDF solution (5 wt % in DMF) was spin-coated onto the dies with a two-step spin-coating protocol (first spin at 1000 rpm for 10 s, second spin at 2000 rpm for 60 s). The PVDF-coated wafers were left to air-dry overnight within the glovebox. Subsequently, they were removed from the glovebox and baked at 120 °C for 2 h to fully remove DMF from the PVDF film. Prior to sensor testing, the embedded MOF layers underwent a final activation step at 150 °C for 3 h in a vacuum oven, ensuring the complete removal of residual solvent.

### 4.9 FET Measurements

Norfentanyl (NF) and dopamine hydrochloride (DA) were individually dissolved in 0.1 M KCl to prepare analyte solutions with concentrations ranging from 1 ppb to 100 ppm. Before measurements, activated sensor devices were incubated in a 0.1 M KCl solution within a 100% humidity chamber for at least 24 h to fully wet the MOF pores. FET transfer characteristics were measured using two Keithley 2400 source-meter units. For each measurement, 300 μL of 0.1 M KCl gating liquid was applied onto the sensor surface. An Ag/AgCl reference electrode, immersed in the gating liquid, served as the gate electrode. A constant bias voltage of 50 mV was applied between the source and drain electrodes, and the gate voltage was swept from +0.6 V to −0.6 V relative to the Ag/AgCl reference electrode. Devices were first stabilized in the 0.1 M KCl gating solution by repeatedly changing and incubating the device in the electrolyte until consistent and stable transfer characteristics were obtained after a 10 min incubation. After achieving a stable baseline, 300 μL of NF or DA analyte solution (prepared in 0.1 M KCl) was introduced as the gating liquid. The device was incubated with each analyte concentration for 10 min before recording FET transfer characteristics. Between measurements of different concentrations, the analyte-containing gating liquid was carefully removed using a pipette, and the subsequent concentration was introduced following the same protocol.

## Supplementary Material



## Notes

Characterization details; SEM and TEM images; XRD characterization; N_2_ sorption isotherms, FET transfer characteristics; and additional calibration plots (PDF)
