# Supplementary material for: Size-Selective FET Sensors Based on Semiconducting Single-Walled Carbon Nanotubes and Metal–Organic Frameworks
Source: ACS Appl Mater Interfaces. 2026 May 18;18(21):30351–60. doi: 10.1021/acsami.6c06870 (PMC13244367; doi:10.1021/acsami.6c06870)
Supplement: Supplementary file 1 [file am6c06870_si_001.pdf]

Supporting Information

Size-Selective FET Sensors Based on Semiconducting Single-Walled Carbon Nanotubes and Metal-Organic Frameworks

*Zidao Zeng[a], Samia Afrin[a], Gefan He[a], Haitao Liu[a], Nathaniel L. Rosi[a,b], Alexander Star[a,c,d]**

[a] Department of Chemistry, University of Pittsburgh, Pittsburgh, Pennsylvania 15260, United States

[b] Department of Chemical & Petroleum Engineering, University of Pittsburgh, Pittsburgh, Pennsylvania 15260, United States

[c] Department of Bioengineering, University of Pittsburgh, Pittsburgh, Pennsylvania 15260, United States

[d] Clinical and Translational Science Institute, University of Pittsburgh, Pittsburgh, Pennsylvania 15260, United States

E-mail: astar@pitt.edu

Table of Contents

Characterization Details……………………………………………………….......................S-4

Figure S1. Photo of the floating single-layer UiO-67 film…………………………..S-5

Figure S2. Top-view SEM images of single-layer UiO-67 with PVDF prepared from different wt% PVDF solution in DMF ....…………………...S-5

Figure S3. Typical I-Vg curves of scSWCNT/UiO-67/PVDF devices fabricated with different PVDF wt% solutions ......…………………………………………………..S-6

Figure S4. Cross-section SEM image of scSWCNT/UiO-67/PVDF ......………………S-7

Figure S5. SEM images of (a) single-layer UiO-67 deposited over scSWCNT, (b) scSWCNT/UiO-67/PVDF.....………………………………………………………..S-8

Figure S6. Top-view SEM image of scSWCNT/UiO-67/PVDF devices fabricated in ambient conditions S-9

Figure S7. TEM images of (a) UiO-66, (b) UiO-67, (c) ZIF-8, and (d) MIL-96……..S-10

Figure S8. N_2 sorption isotherm at 77 K for UiO-66, UiO-67, ZIF-8, and MIL-96......S-11

Figure S9. Relative response of scSWCNT/UiO-67/PVDF devices at different gate voltage when exposed to norfentanyl S-12

Figure S10. I_{ds}-V_g characteristic of a typical scSWCNT/UiO-67 device ....…........S-13

Figure S11. Comparison of the sensing responses toward norfentanyl for scSWCNT/UiO-67/PVDF devices fabricated using PVDF solutions with different weight percentages ....….....…………………………………………………………..S-14

Figure S12. Cyclic voltammogram of scSWCNT, scSWCNT/UiO-67, and scSWCNT/UiO-67/PVDF...S-15

Figure S13. Normalized capacitance at −0.2 V (vs Ag/AgCl) for three sensor architectures: scSWCNT, scSWCNT/UiO-67, and scSWCNT/UiO-67/PVDF as a function of norfentanyl concentration in the electrolyte..S-16

Figure S14. Relative response of a) scSWCNT/UiO-66/PVDF, b) scSWCNT/UiO-67/PVDF c) scSWCNT/MIL-96/PVDF devices at different gate voltages when exposed to dopamine...S-17

Figure S15. Calibration plots with linear fits of scSWCNT/MOF/PVDF devices for norfentanyl and dopamine detection S-18

Figure S16. SEM images of transferred single-layer MOF…………………………...S-19

Figure S17. I_{ds}-V_g characteristics for devices fabricated with (a) UiO-66, (b) ZIF-8, and (c) MIL-96 (norfentanyl) ……….....…………………………………………………. S-20

Figure S18. Calibration plot of devices fabricated with scSWCNT and MOFs…...….S-21

Figure S19. Comparison of sensor responses between standard scSWCNT/UiO-67/PVDF devices and those with five additional UiO-67 MOF layers …...……………………...S-22

Figure S20. I_{ds}-V_g characteristics for typical scSWCNT/MOF/PVDF devices fabricated with (a) UiO-66, (b) UiO-67, (c) ZIF-8, and (d) MIL-96 (dopamine)………...………S-23

Figure S21. Top-view SEM image of scSWCNT/MOF/PVDF device after dopamine testing S-24

Figure S22. XRD pattern of scSWCNT/MOF/PVDF devices after dopamine test S-25

Figure S23. Calibration plots of scSWCNT/MOF/PVDF devices toward dopamine after storing in 0.1 M KCl for two months S-26

Figure S24. Top-view SEM images of scSWCNT/MOF/PVDF devices after storing in 0.1 M KCl for two months …………………………...............................……S-27

Figure S25. Comparison of sensor response of scSWCNT/UiO-67/PVDF devices toward norfentanyl in 0.1 M KCl, synthetic urine, and BSA solutions ……………………………..........................……………………………S-28

Table S1. Comparison of published norfentanyl detection methods S-29

Table S2. Comparison of published electrochemical dopamine detection methods S-30

References...S-31

Characterization Details

Transmission Electron Microscopy: Transmission electron microscopy was performed on a Hitachi 7800 instrument operating at 80 kV.

Powder X-ray diffraction: PXRD was conducted with Bruker D8 XRD system equipped with LynxEye detector. Samples were prepared by drop-casting on glass slides. Diffraction patterns were collected over a 2θ range from 3.5° to 50°, with a step size of 0.02° and an acquisition time of 0.3 seconds per step. The X-ray source was Cu Kα operating at 40 kV and 40 μA.

Scanning electron microscopy: Scanning electron microscopy was performed on a Zeiss Sigma 500 VP analytical FE-SWM instrument operating at 1 kV. Non-conductive samples were coated with a thin layer of PdAu alloy (~3nm) using a Denton Sputter Coater.

N_2 adsorption isotherms: Gas adsorption isotherms were collected on a Micromeritics 3-flex gas adsorption analyzer. Approximately 60 mg of each sample was added into a pre-weighed sample analysis tube that had been evacuated and backfilled with N_2 before massing. The samples were degassed at 423 K under vacuum for 24 hours until the pressure change rate was no more than 3.5 mTorr/min. A liquid N_2 bath was used for the N_2 adsorption experiments at 77 K. UHP grade N_2 adsorbate (99.999 %) was used in this study.

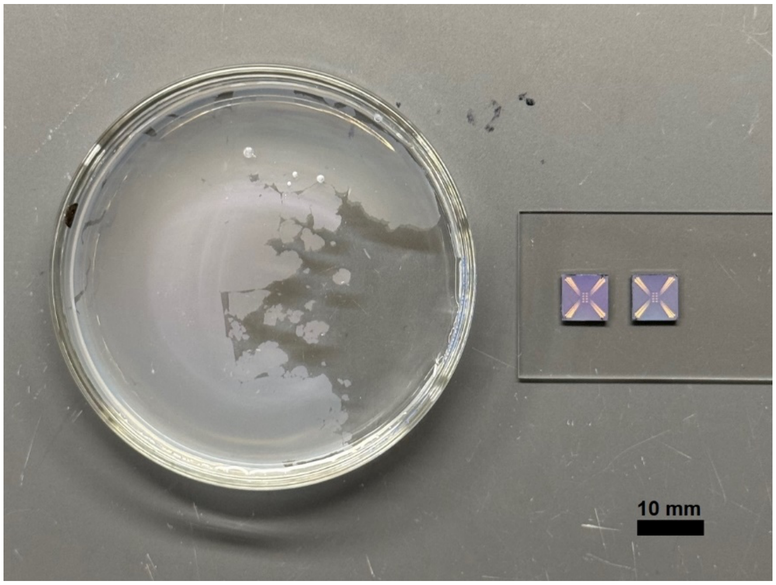

Figure S1. Photo of the floating single-layer UiO-67 film on water surface in a petri dish and silicon wafer dies (7 × 7 mm) with transferred UiO-67 film.

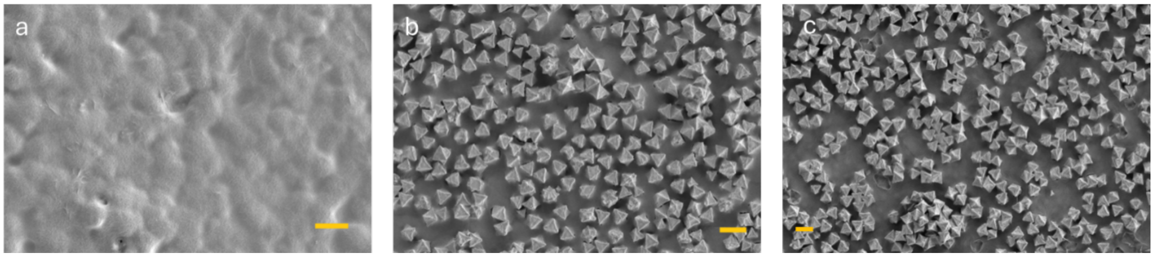

Figure S2. Top-view SEM images of single-layer UiO-67 with PVDF prepared from different wt% PVDF solution in DMF. (a) 7.5 wt%, (b) 5 wt%, and (c) 2.5 wt%. Scale bars are 1 μm.

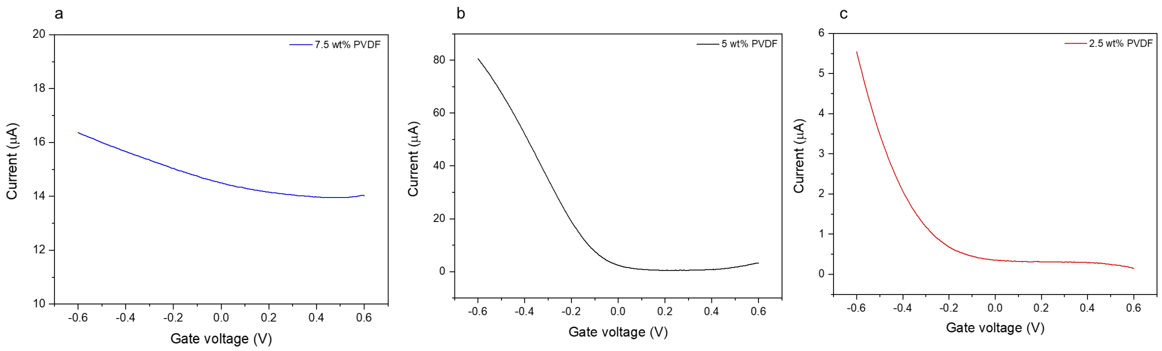

Figure S3. Typical I-Vg curves of scSWCNT/UiO-67/PVDF devices fabricated with different PVDF wt% solutions. (a) 7.5 wt%, (b) 5 wt%, and (c) 2.5 wt%. Gating liquid is 0.1 M KCl.

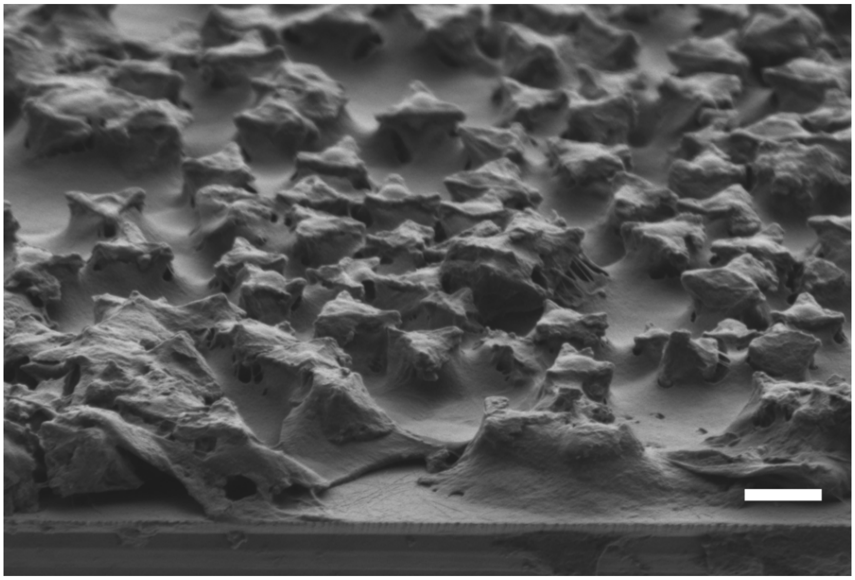

Figure S4. Cross-section SEM image of scSWCNT/UiO-67/PVDF. The sample was coated with Pd-Au alloy. The scale bar is 1 μm.

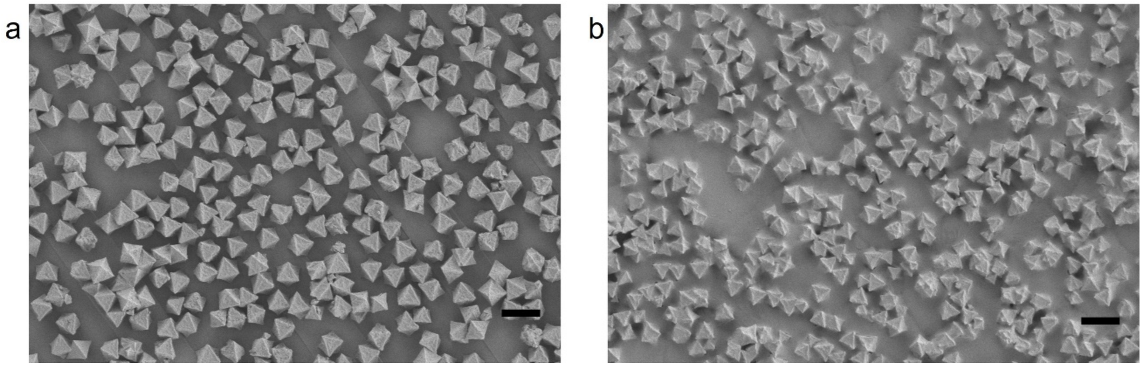

Figure S5. SEM images of (a) single-layer UiO-67 deposited over scSWCNT, (b) scSWCNT/UiO-67/PVDF. Scale bars are 2 μm.

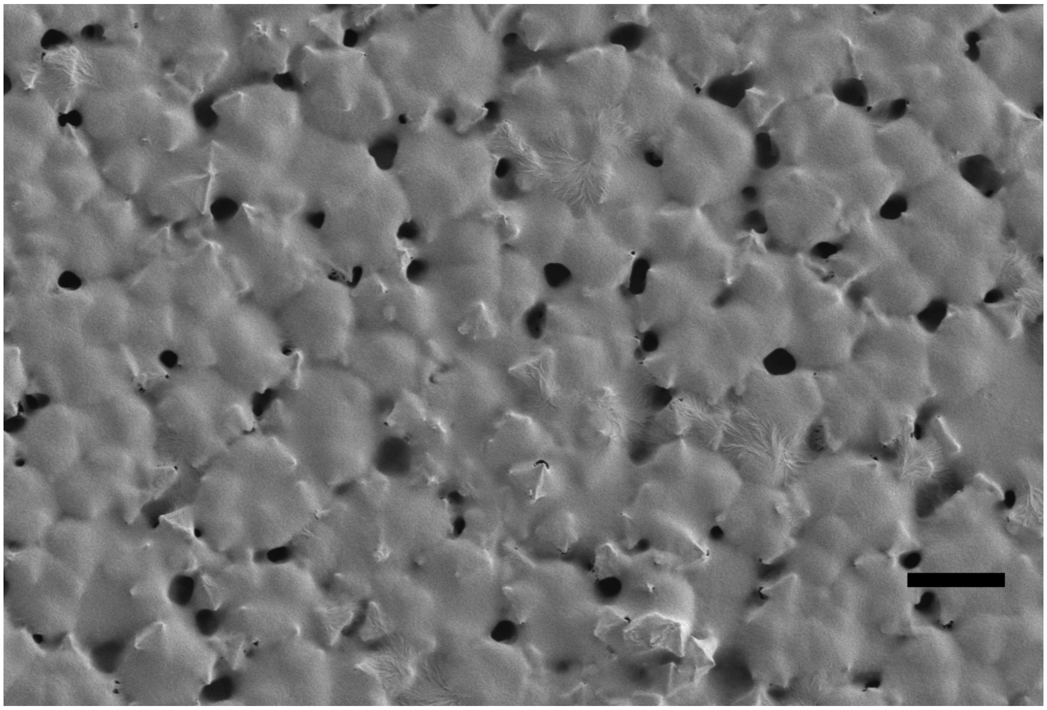

Figure S6. Top-view SEM image of scSWCNT/UiO-67/PVDF devices fabricated in ambient conditions. The black holes are the macrovoids resulted from adsorbed water in air. The scale bar is 3 μm.

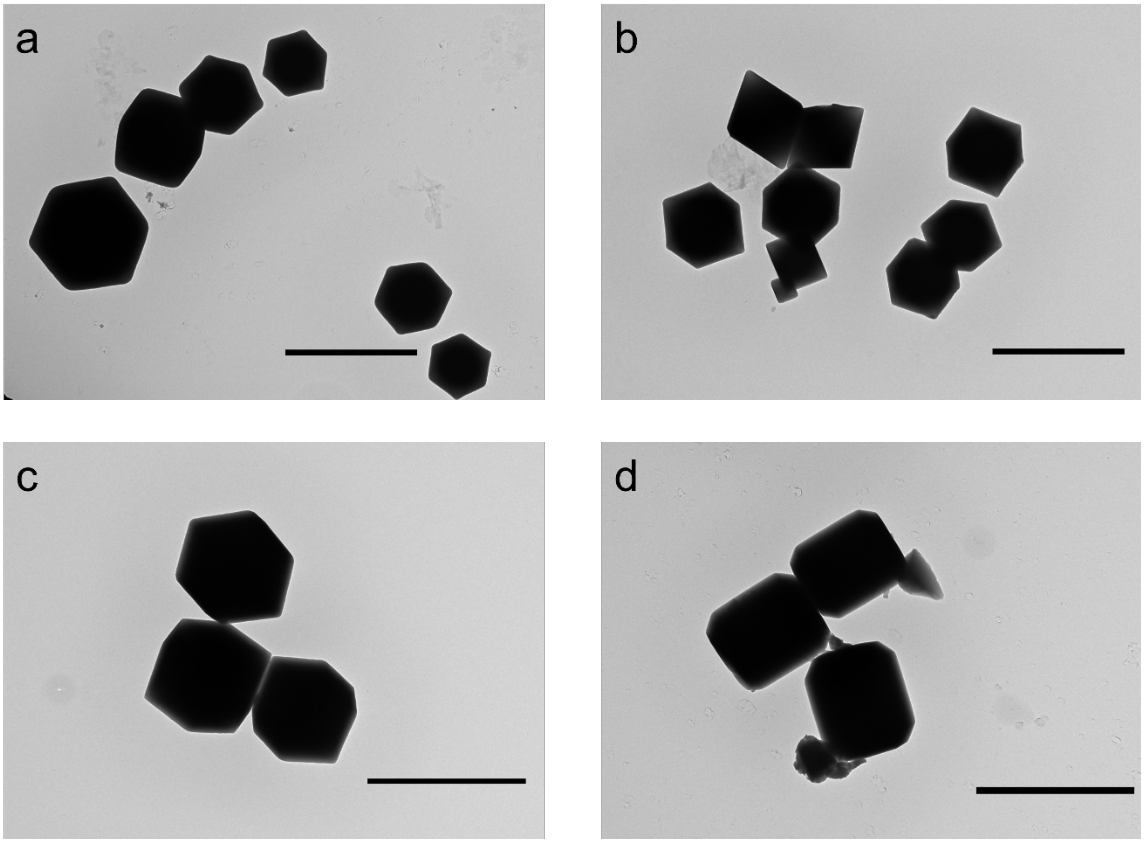

Figure S7. TEM images of (a) UiO-66, (b) UiO-67, (c) ZIF-8, and (d) MIL-96. Scale bars are 2 μm.

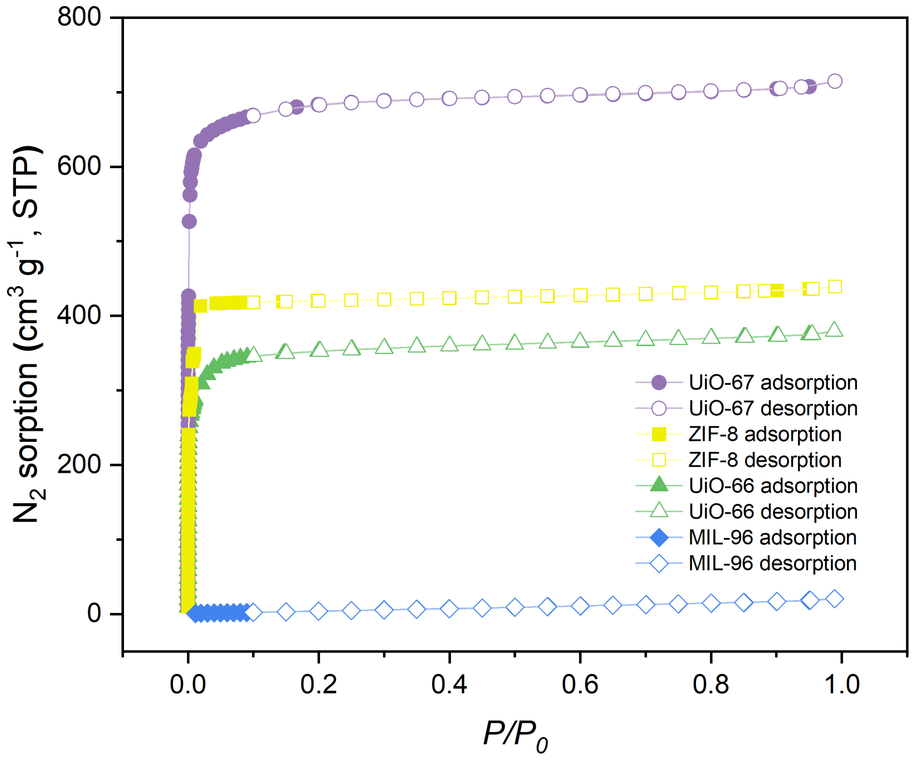

Figure S8. N_2 sorption isotherm at 77 K for UiO-66 (green), UiO-67 (purple), ZIF-8 (yellow), and MIL-96 (blue).

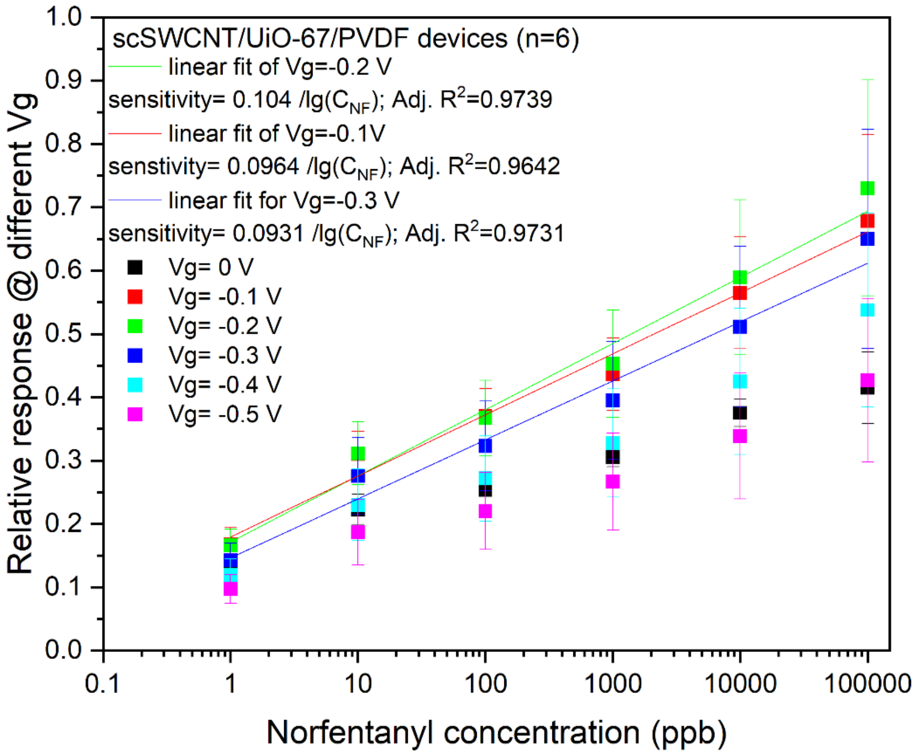

Figure S9. Relative response of scSWCNT/UiO-67/PVDF devices at different gate voltage when exposed to norfentanyl.

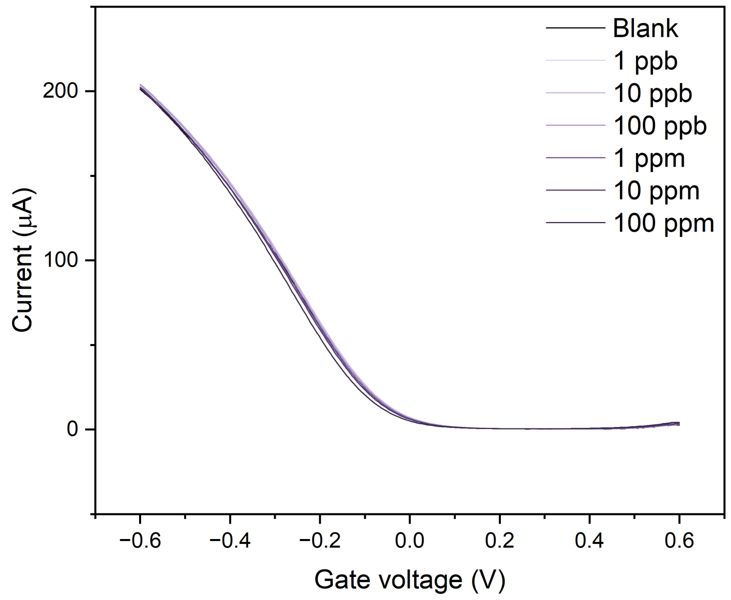

Figure S10. I_{ds}-V_g characteristic of a typical scSWCNT/UiO-67 device when exposed to norfentanyl at different concentration.

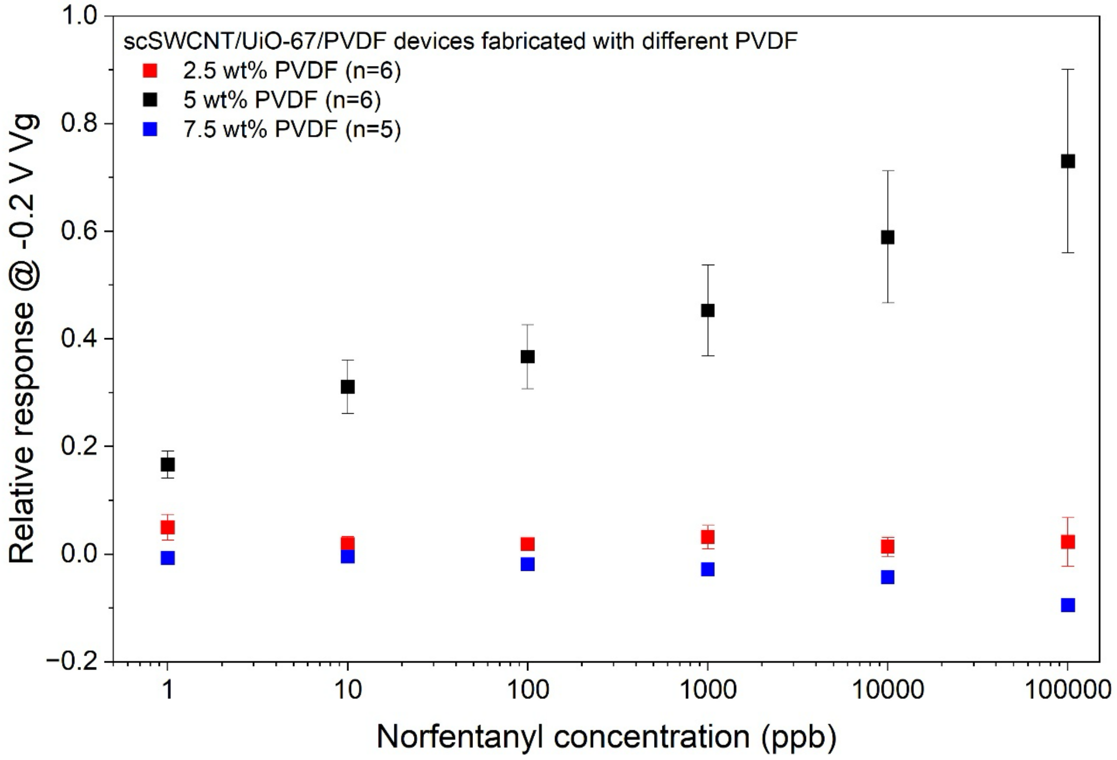

Figure S11. Comparison of the sensing responses toward norfentanyl for scSWCNT/UiO-67/PVDF devices fabricated using PVDF solutions with different weight percentages.

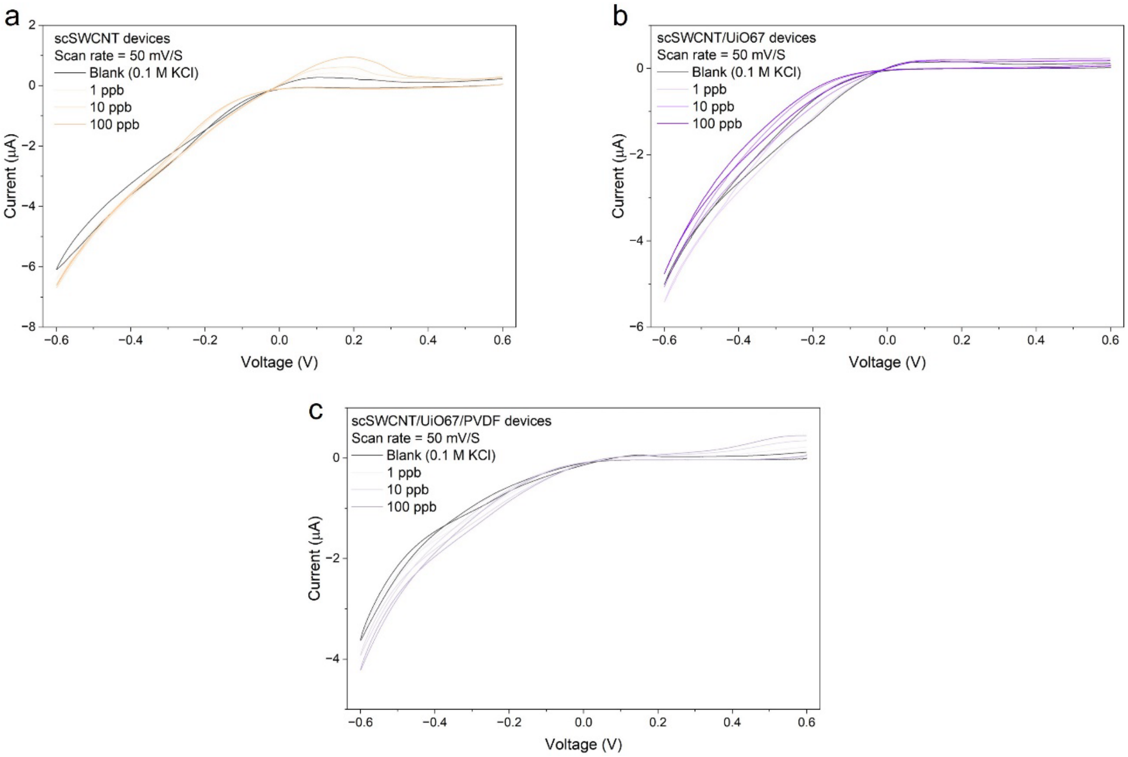

Figure S12. Cyclic voltammograms of (a) scSWCNT, (b) scSWCNT/UiO-67, and (c) scSWCNT/UiO-67/PVDF with Ag/AgCl reference electrode. 0.1 M KCl solutions with and without norfentanyl were used as electrolyte. Scan rate is 50 mV s^{-1}.

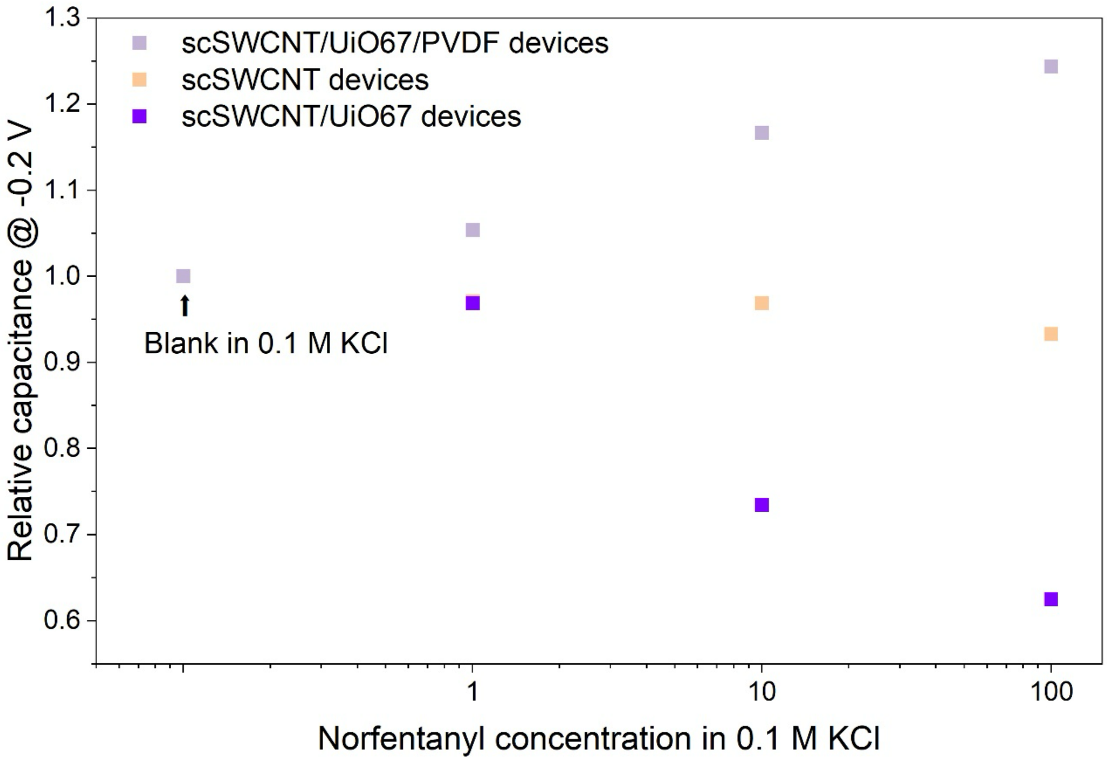

Figure S13. Normalized capacitance at −0.2 V (vs Ag/AgCl) for three sensor architectures: scSWCNT, scSWCNT/UiO-67, and scSWCNT/UiO-67/PVDF as a function of norfentanyl concentration in the electrolyte.

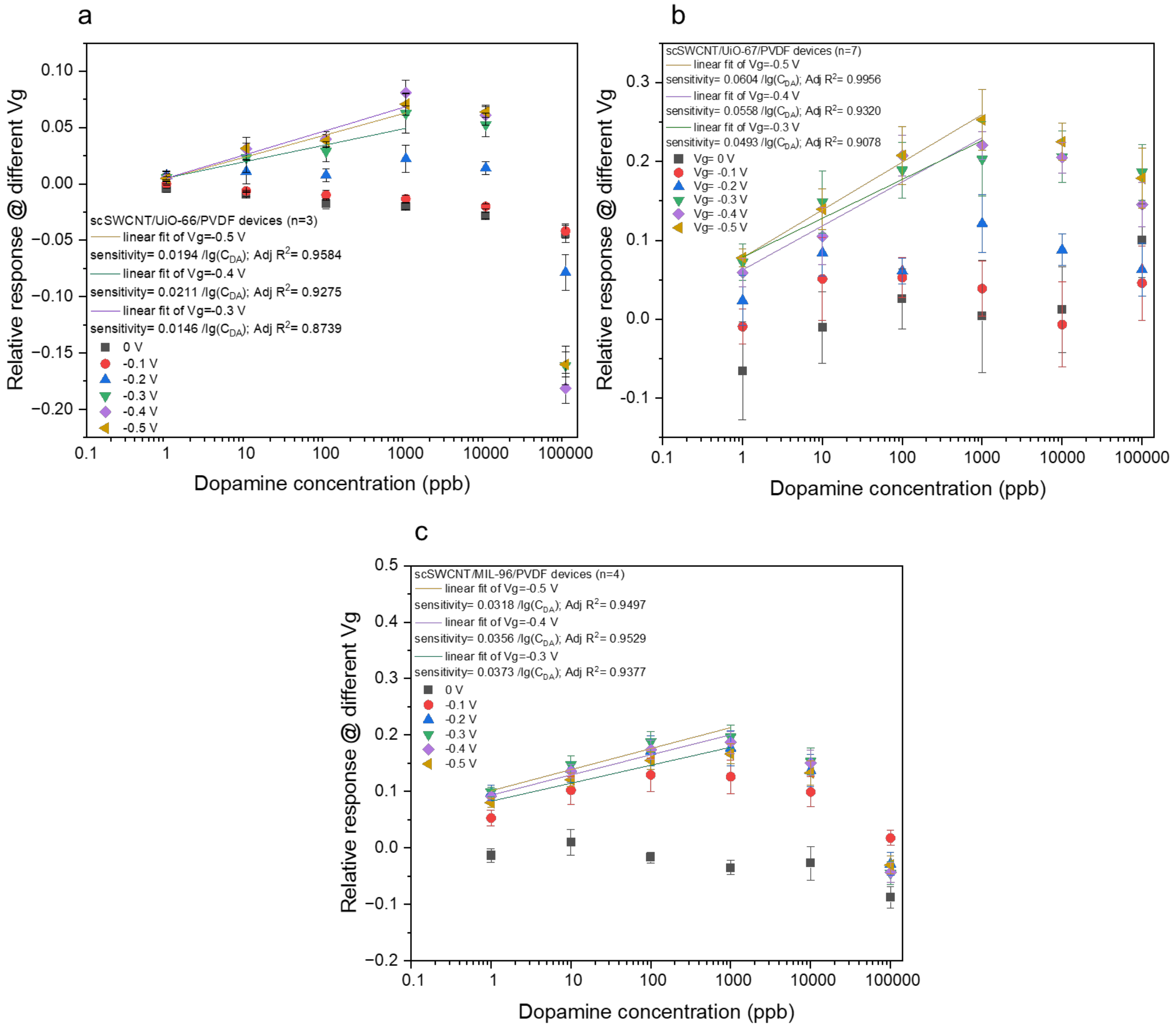

Figure S14. Relative response of a) scSWCNT/UiO-66/PVDF, b) scSWCNT/UiO-67/PVDF c) scSWCNT/MIL-96/PVDF devices at different gate voltages when exposed to dopamine.

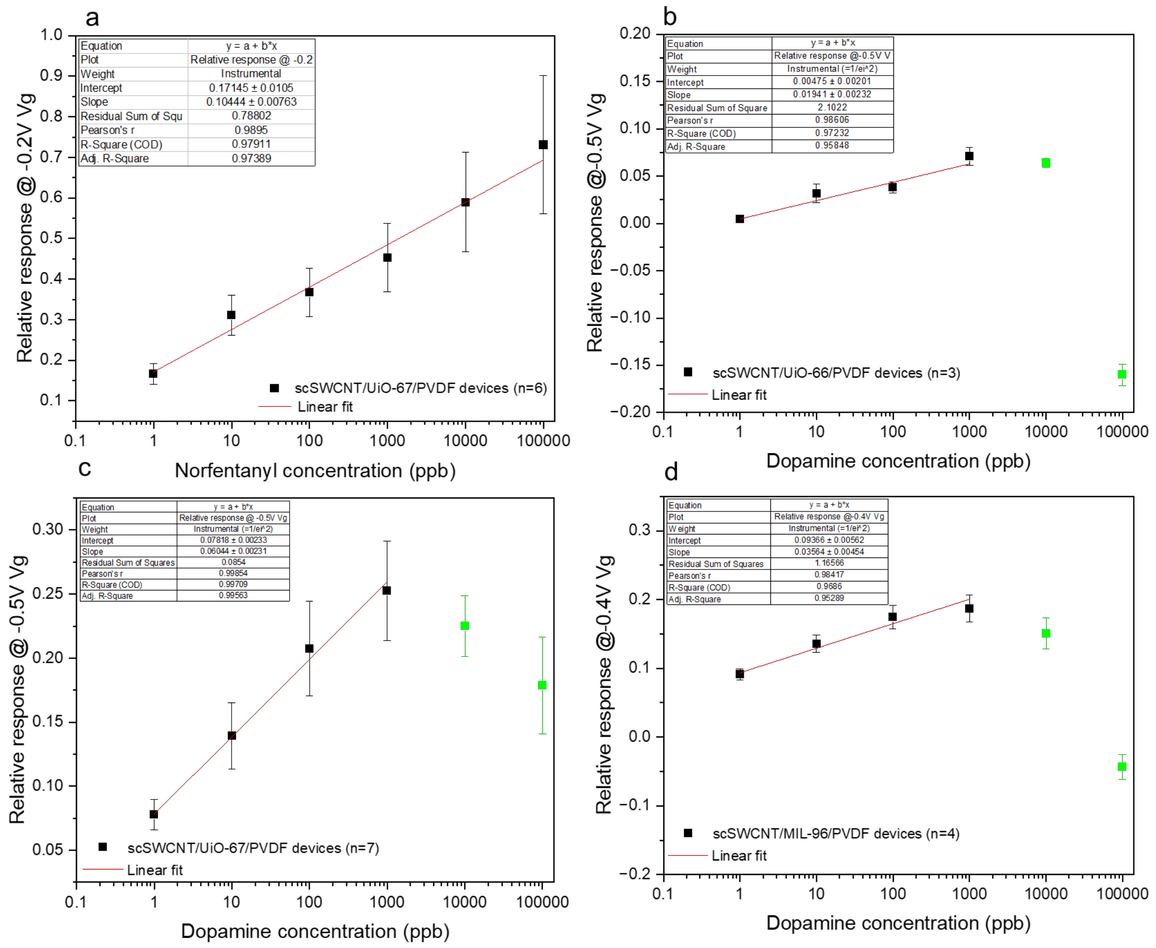

Figure S15. Calibration plots with linear fits of scSWCNT/MOF/PVDF devices for norfentanyl and dopamine detection. (a) scSWCNT/UiO-67/PVDF devices toward norfentanyl. (b) scSWCNT/UiO-66/PVDF, (c) scSWCNT/UiO-67/PVDF, and (d) scSWCNT/MIL-96/PVDF devices toward dopamine. Linear fitting was restricted to consistent current increase range for capacitance modulation mechanism.

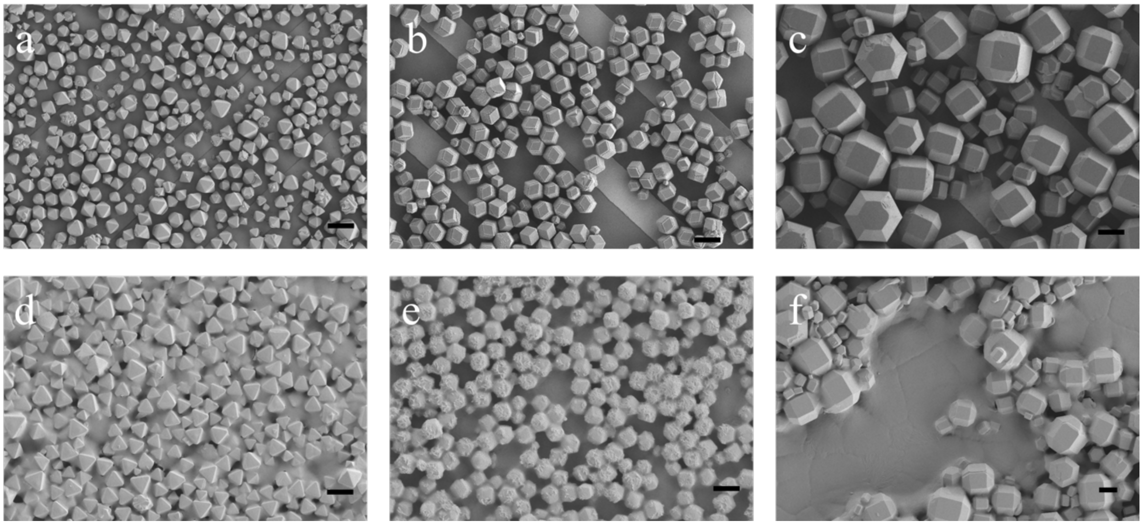

Figure S16. SEM images of transferred single-layer MOF on silicon dies (a-c) and devices coated with PVDF (d-f). (a) and (d) are UiO-66, (b) and (e) are ZIF-8, (c) and (f) are MIL-96. The underling strip patterns are the gold interdigitated electrodes. Scale bars are 2 μm.

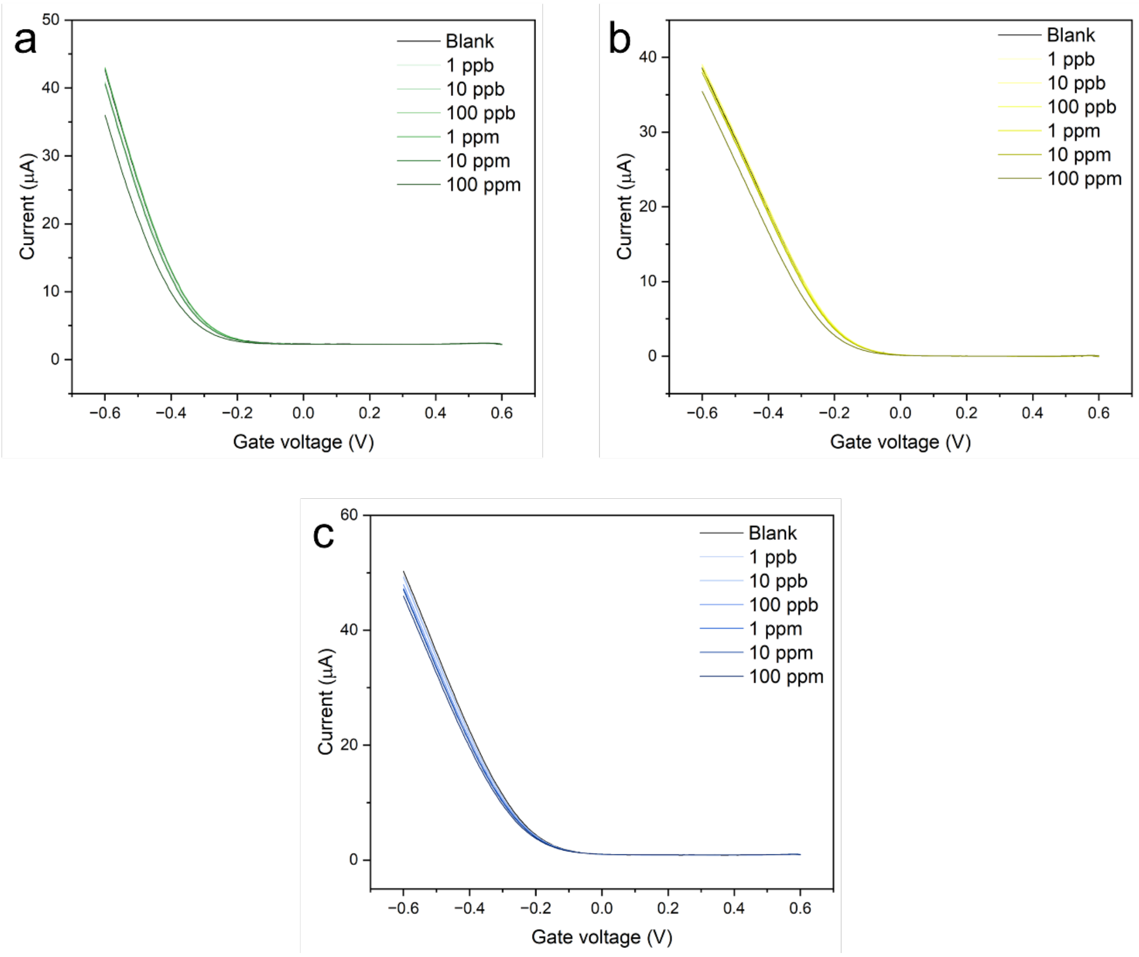

Figure S17. I_{ds}-V_g characteristics for devices fabricated with (a) UiO-66, (b) ZIF-8, and (c) MIL-96, when exposed to norfentanyl in 0.1 M KCl.

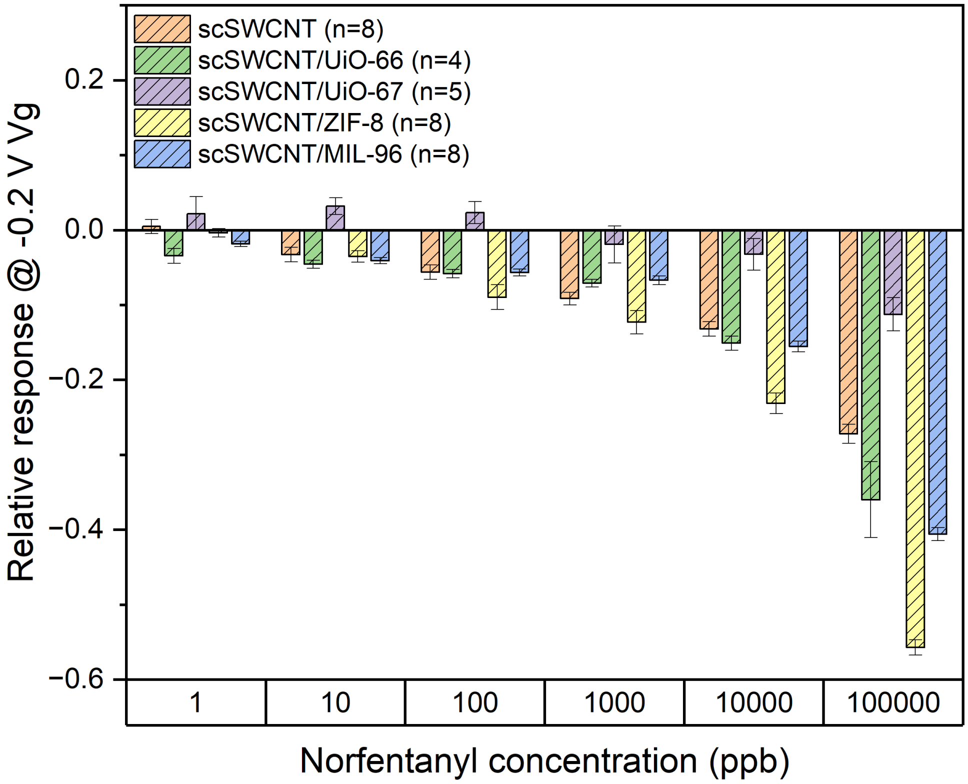

Figure S18. Calibration plot of devices fabricated with scSWCNT and MOFs toward norfentanyl at different concentration in 0.1 M KCl, no PVDF was coated.

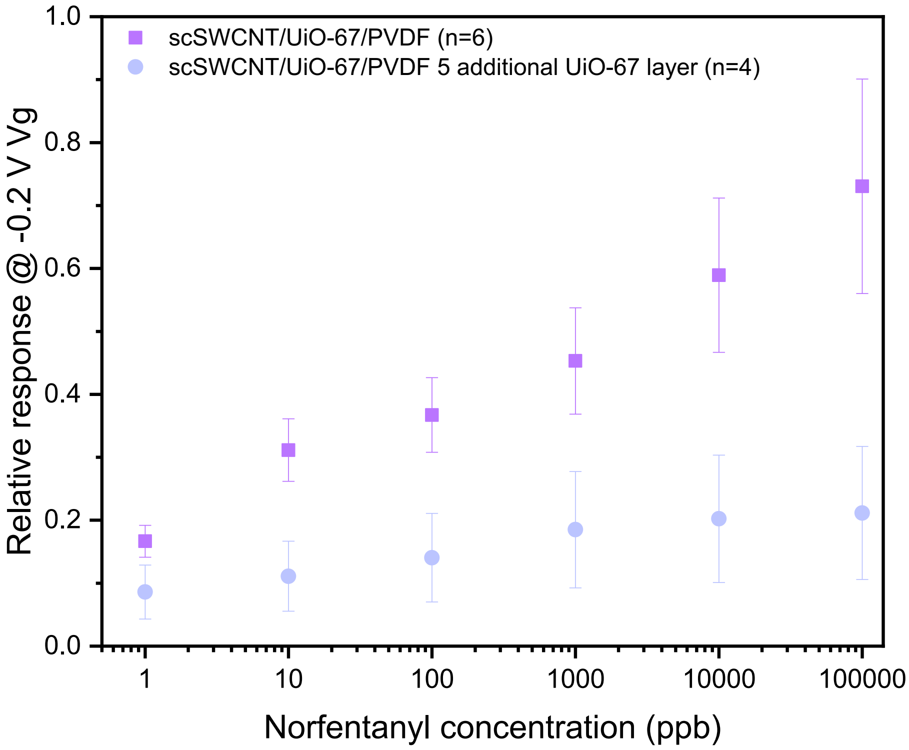

Figure S19. Comparison of sensor responses between standard scSWCNT/UiO-67/PVDF devices and those with five additional UiO-67 MOF layers.

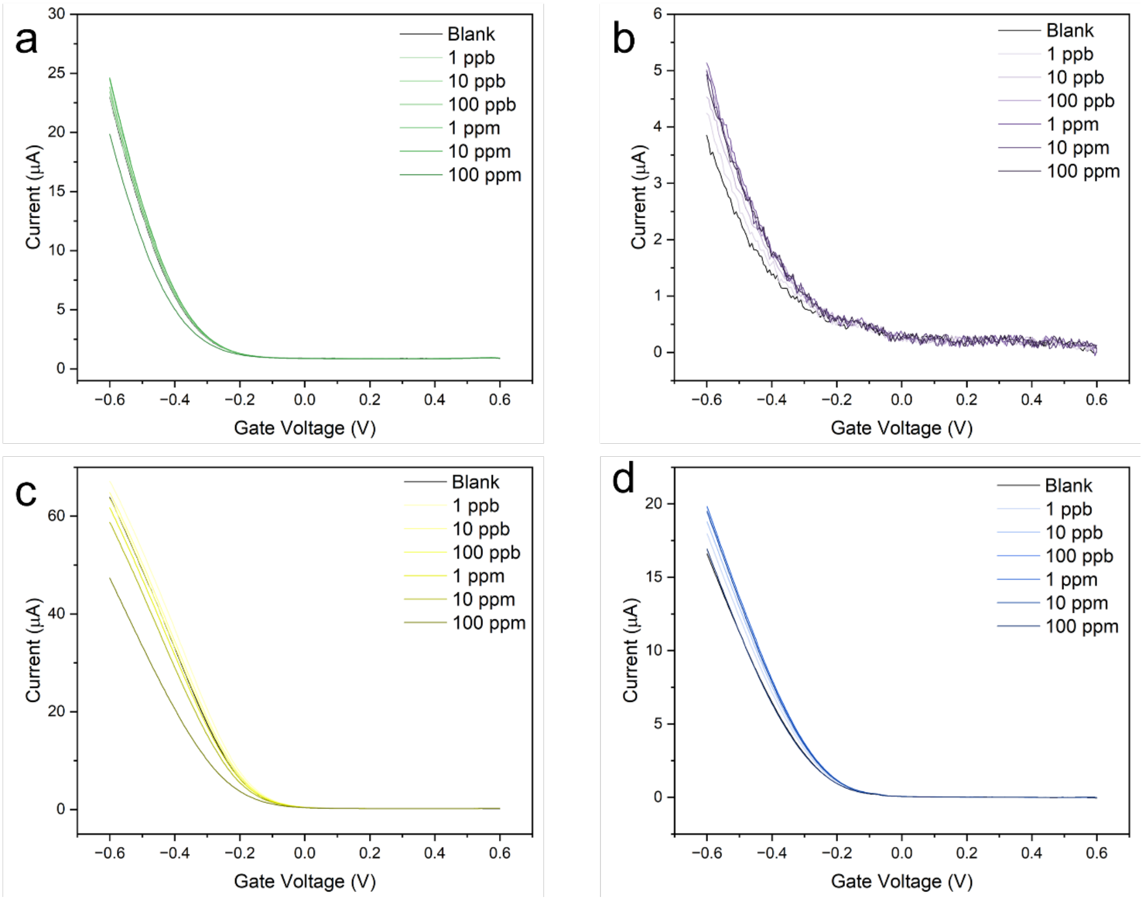

Figure S20. I_{ds}-V_g characteristics for typical scSWCNT/MOF/PVDF devices fabricated with (a) UiO-66, (b) UiO-67, (c) ZIF-8, and (d) MIL-96 when exposed to dopamine at different concentration in 0.1 M KCl.

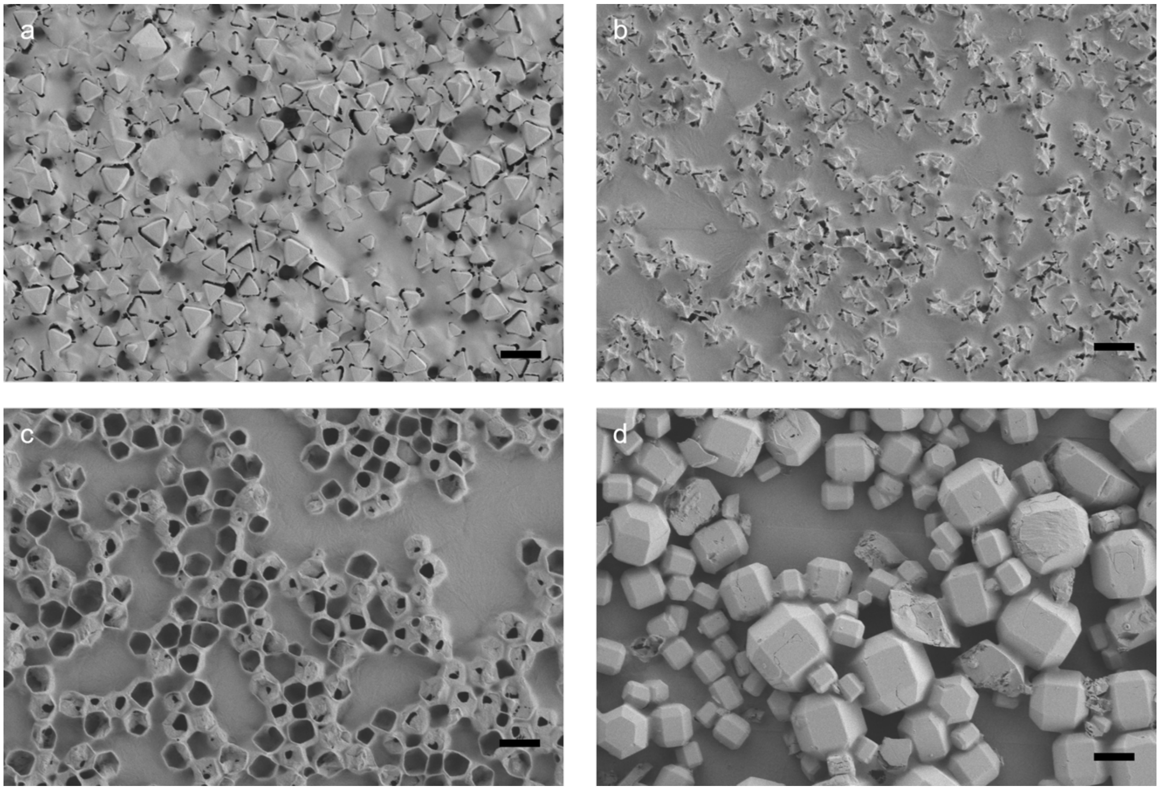

Figure S21. Top-view SEM image of scSWCNT/MOF/PVDF device after dopamine testing. (a) scSWCNT/UiO-66/PVDF device. (b) scSWCNT/UiO-67-PVDF device. (c) scSWCNT/ZIF-8/PVDF device. (d) scSWCNT/MIL-96/PVDF devices. The scale bars are2 μm.

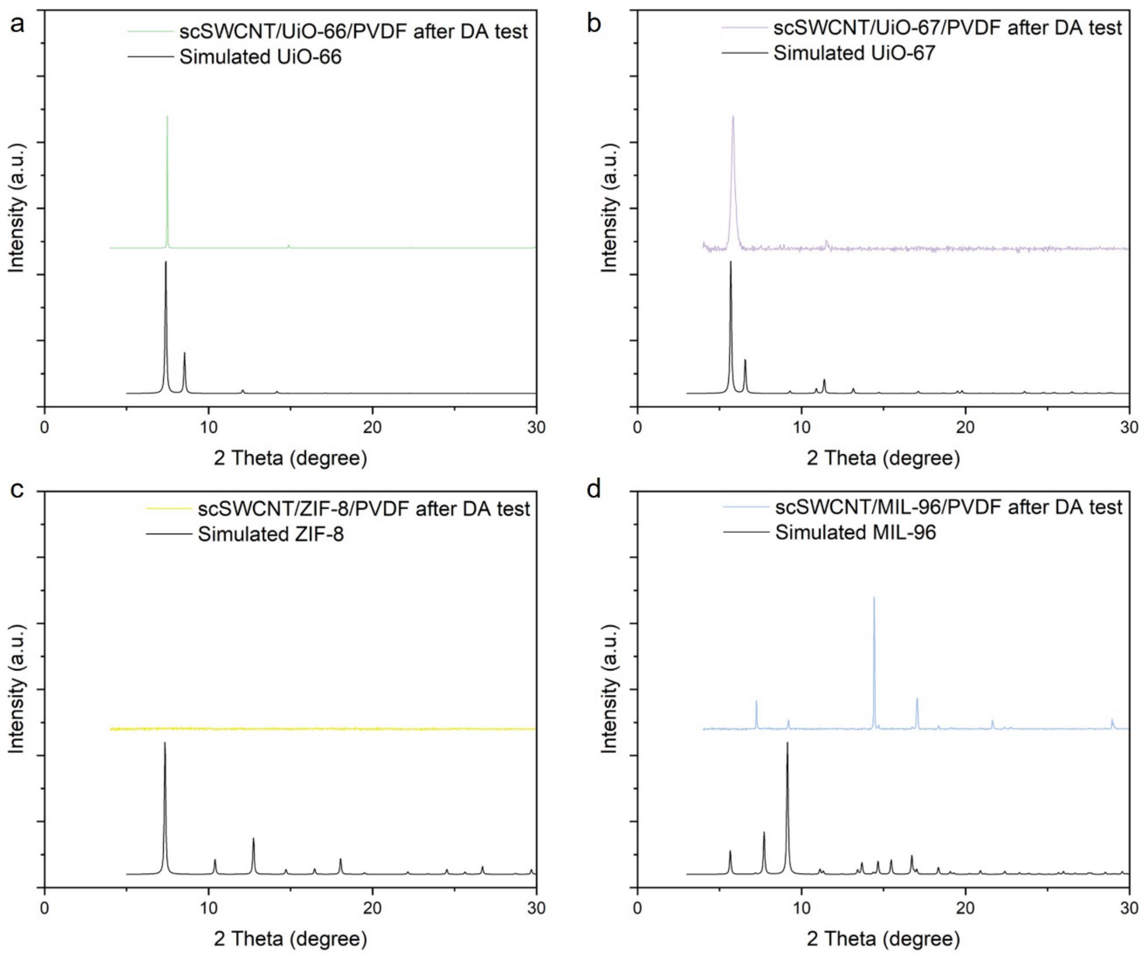

Figure S22. XRD pattern of scSWCNT/MOF/PVDF devices after dopamine test. (a) UiO-66 devices. (b) UiO-67 devices. (c) ZIF-8 devices. (d) MIL-96 devices.

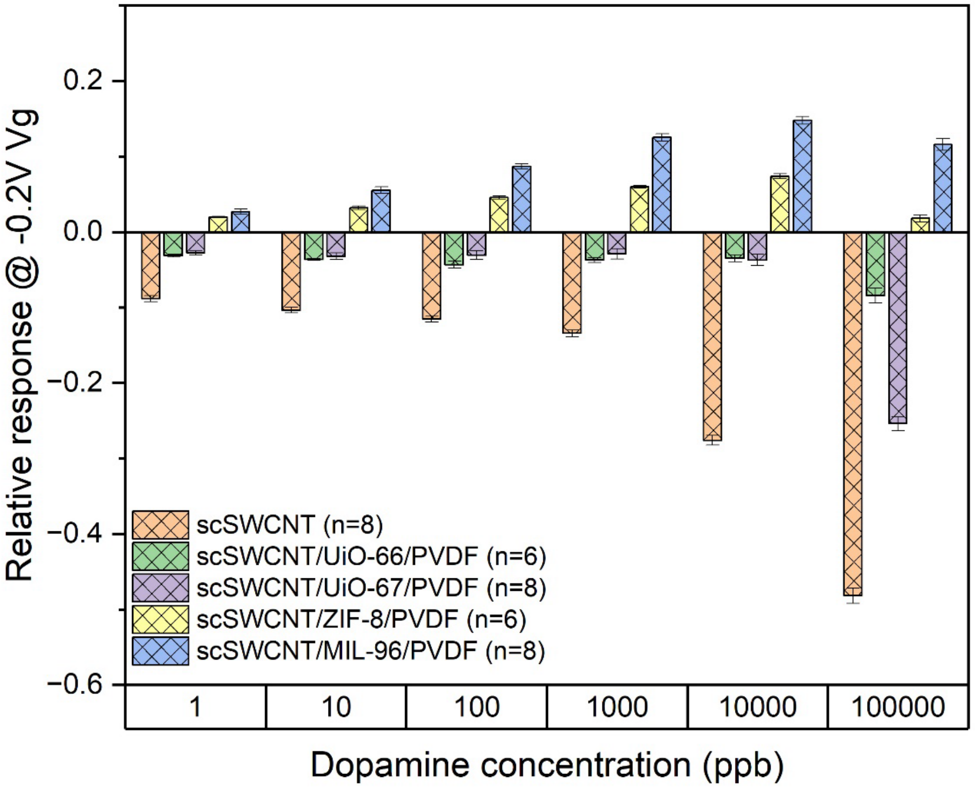

Figure S23. Calibration plots of scSWCNT/MOF/PVDF devices toward dopamine at different concentrations in 0.1 M KCl after storing in KCl solution for 2 months.

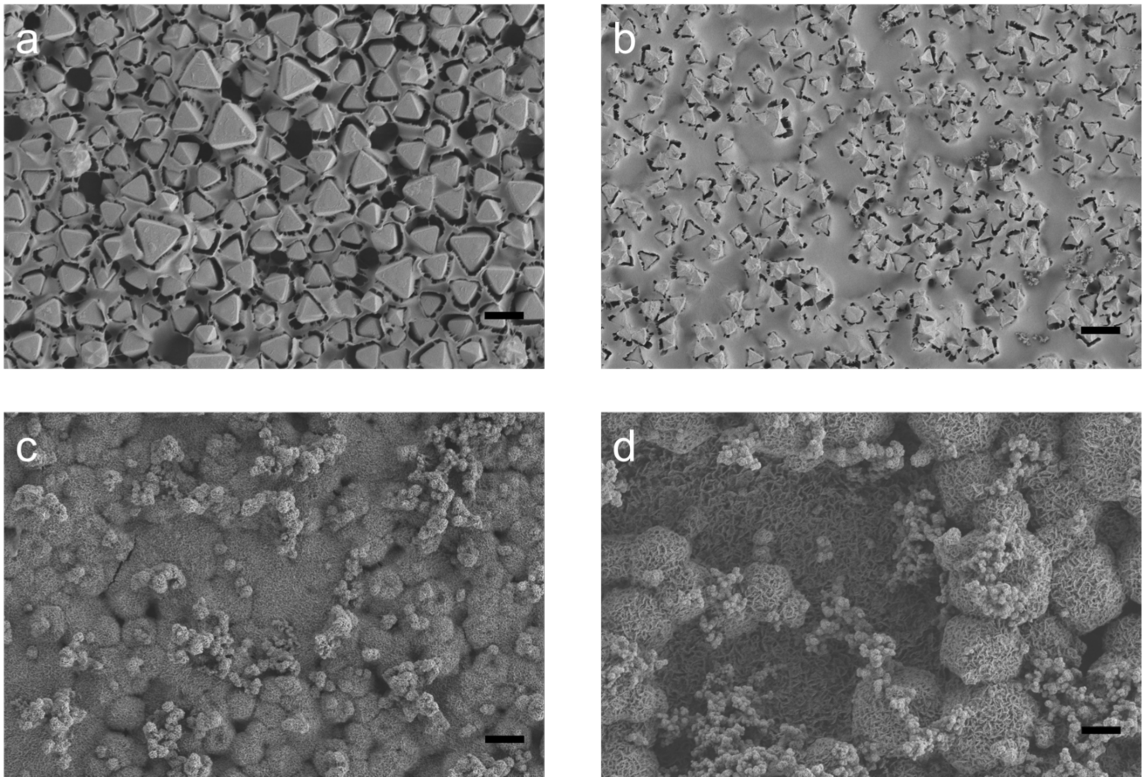

Figure S24. Top-view SEM images of scSWCNT/MOF/PVDF devices fabricated with (a) UiO-66, (b) UiO-67, (c) ZIF-8, and (d) MIL-96 after storing in 0.1 M KCl for two months. Scale bars are 2 μm.

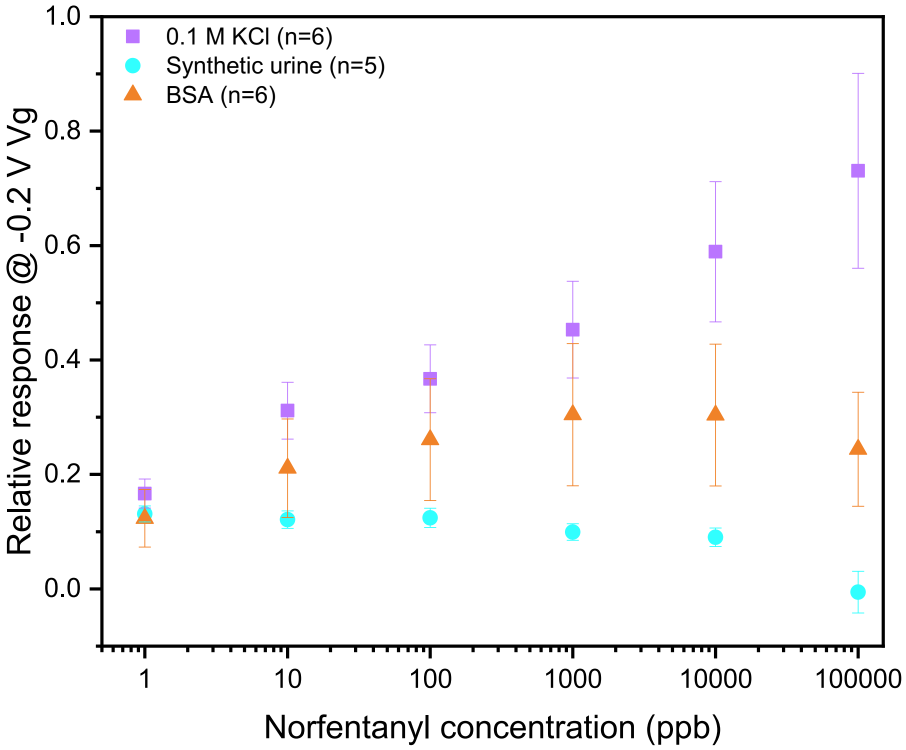

Figure S25. Comparison of sensor response of scSWCNT/UiO-67/PVDF devices toward norfentanyl in 0.1 M KCl, synthetic urine, and BSA solutions.

Table S1. Comparison of published norfentanyl detection methods.

Sensing method	Limit of detection	Sample source	Reference
LFIA	8 ng/mL	urine	1
LFIA	100 ng/mL	saliva	1
G-FET	42 pg/mL	wastewater diluted in PBS	2
sc-SWCNT-ab-FET	2.0 fg/mL<br>3.7 fg/mL	synthetic urine diluted in PBS	3
SWCNT@MOF-FET	48 ng/mL	PBS	4
Fluorescent prob	0.3 pg/mL	aqueous solution	5
SWCNT/MOF/PVDF-FET	12 ng/mL	KCl solution	this work

Table S2. Comparison of published electrochemical dopamine detection methods.

Sensing method	Limit of detection	Sample source	Reference
Try@PANI-CNTs-CNC<br>CV	0.24 ppb	aqueous sample	6
Tyr-LIMG<br>DPV	15 ppt	urine	7
Apt-GCSC-GO/GCE<br>EIS	0.11 ppb	aqueous sample	8
AuNPs@MXene/SPGE<br>CV/DPV	0.44 ppb	PBS	9
Fe3O4/GCE<br>Ampe	0.12 ppb	PBS	10
AgNps-CuO/ ITO<br>CV	1.1 ppb	PBS	11
MIPAA-BiVO4/GCE<br>Ampe	0.44 ppb	PBS	12
MIP-MWCNTs- GAs/GCE<br>DPV	0.26 ppb	PBS	13
scSWCNT/MOF/PVDF<br>FET	14 ppb<br>9ppb<br>11ppb	KCl	this work

References

1. Boone, P.; He, Y.; Lieber, A. R.; Steckel, J. A.; Rosi, N. L.; Hornbostel, K. M.; Wilmer, C. E., Designing optimal core-shell MOFs for direct air capture. *Nanoscale* **2022,** *14* (43), 16085-16096.
2. Kumar, N.; Rana, M.; Geiwitz, M.; Khan, N. I.; Catalano, M.; Ortiz-Marquez, J. C.; Kitadai, H.; Weber, A.; Dweik, B.; Ling, X.; van Opijnen, T.; Argun, A. A.; Burch, K. S., Rapid, Multianalyte Detection of Opioid Metabolites in Wastewater. *ACS Nano* **2022,** *16* (3), 3704-3714.
3. Shao, W.; Zeng, Z.; Star, A., An Ultrasensitive Norfentanyl Sensor Based on a Carbon Nanotube-Based Field-Effect Transistor for the Detection of Fentanyl Exposure. *ACS Appl Mater Interfaces* **2023,** *15* (31), 37784-37793.
4. Zeng, Z.; Islamov, M.; He, Y.; Day, B. A.; Rosi, N. L.; Wilmer, C. E.; Star, A., Size-Based Norfentanyl Detection with SWCNT@UiO-MOF Composites. *ACS Appl Mater Interfaces* **2024,** *16* (1), 1361-1369.
5. Sun, J.; Zhi, J.; Zhang, L.; Qi, Y.; Sun, J.; Jin, Y.; Yin, J.; Yao, K.; Shao, B., An "On-Off" AIE-Based Lock-and-Key Fluorescent Probe System for Detection of Fentanyl/Norfentanyl. *Molecules* **2025,** *30* (9).
6. Dhanjai; Yu, N.; Mugo, S. M., Disposable Capacitive Biosensor for Dopamine Sensing. *ChemistrySelect* **2020,** *5* (40), 12470-12476.
7. Suriyaprakash, J.; Huang, Y.; Hu, Z.; Wang, H.; Zhan, Y.; Zhou, Y.; Thangavelu, I.; Wu, L., Laser Scribing Turns Plastic Waste into a Biosensor via the Restructuration of Nanocarbon Composites for Noninvasive Dopamine Detection. *Biosensors (Basel)* **2023,** *13* (8).
8. Wei, B.; Zhong, H.; Wang, L.; Liu, Y.; Xu, Y.; Zhang, J.; Xu, C.; He, L.; Wang, H., Facile preparation of a collagen-graphene oxide composite: A sensitive and robust electrochemical aptasensor for determining dopamine in biological samples. *Int J Biol Macromol* **2019,** *135*, 400-406.
9. Duan, H.; Tang, S. Y.; Goda, K.; Li, M., Enhancing the sensitivity and stability of electrochemical aptamer-based sensors by AuNPs@MXene nanocomposite for continuous monitoring of biomarkers. *Biosens Bioelectron* **2024,** *246*, 115918.
10. Huang, Y.; Zhang, Y.; Liu, D.; Li, M.; Yu, Y.; Yang, W.; Li, H., Facile synthesis of highly ordered mesoporous Fe(3)O(4) with ultrasensitive detection of dopamine. *Talanta* **2019,** *201*, 511-518.
11. Li, Y.-Y.; Kang, P.; Wang, S.-Q.; Liu, Z.-G.; Li, Y.-X.; Guo, Z., Ag nanoparticles anchored onto porous CuO nanobelts for the ultrasensitive electrochemical detection of dopamine in human serum. *Sensors and Actuators B: Chemical* **2021,** *327*.
12. Gorla, F. A.; Santana Dos Santos, C.; de Matos, R.; Antigo Medeiros, R.; do Prado Ferreira, M.; Pereira Camargo, L.; Henrique Dall'Antonia, L.; Tarley, C. R. T., Development of novel nanocomposite-modified photoelectrochemical sensor based on the association of bismuth vanadate and MWCNT-grafted-molecularly imprinted poly(acrylic acid) for dopamine determination at nanomolar level. *Talanta* **2024,** *266* (Pt 2), 125044.

13. Ma, X.; Gao, F.; Dai, R.; Liu, G.; Zhang, Y.; Lu, L.; Yu, Y., Novel electrochemical sensing platform based on a molecularly imprinted polymer-decorated 3D-multi-walled carbon nanotube intercalated graphene aerogel for selective and sensitive detection of dopamine. *Analytical Methods* **2020,** *12* (14), 1845-1851.
